# Genome-wide association studies and genomic prediction of breeding values for calving performance and body conformation traits in Holstein cattle

**DOI:** 10.1186/s12711-017-0356-8

**Published:** 2017-11-07

**Authors:** Mohammed K. Abo-Ismail, Luiz F. Brito, Stephen P. Miller, Mehdi Sargolzaei, Daniela A. Grossi, Steve S. Moore, Graham Plastow, Paul Stothard, Shadi Nayeri, Flavio S. Schenkel

**Affiliations:** 1 Department of Animal Biosciences, Centre for Genetic Improvement of Livestock, University of Guelph, Guelph, ON Canada; 2 Department of Animal and Poultry Production, Damanhour University, Damanhour, Egypt; 3 The Angus Genetics Inc, Saint Joseph, MO USA; 4 The Semex Alliance, Guelph, ON Canada; 5 University of Queensland, Brisbane, QLD Australia; 6 University of Alberta, Edmonton, AB Canada

## Abstract

**Background:**

Our aim was to identify genomic regions via genome-wide association studies (GWAS) to improve the predictability of genetic merit in Holsteins for 10 calving and 28 body conformation traits. Animals were genotyped using the Illumina Bovine 50 K BeadChip and imputed to the Illumina BovineHD BeadChip (HD). GWAS were performed on 601,717 real and imputed single nucleotide polymorphism (SNP) genotypes using a single-SNP mixed linear model on 4841 Holstein bulls with breeding value predictions and followed by gene identification and in silico functional analyses. The association results were further validated using five scenarios with different numbers of SNPs.

**Results:**

Seven hundred and eighty-two SNPs were significantly associated with calving performance at a genome-wise false discovery rate (FDR) of 5%. Most of these significant SNPs were on chromosomes 18 (71.9%), 17 (7.4%), 5 (6.8%) and 7 (2.4%) and mapped to 675 genes, among which 142 included at least one significant SNP and 532 were nearby one (100 kbp). For body conformation traits, 607 SNPs were significant at a genome-wise FDR of 5% and most of them were located on chromosomes 5 (30%), 18 (27%), 20 (13%), 6 (6%), 7 (5%), 14 (5%) and 13 (3%). SNP enrichment functional analyses for calving traits at a FDR of 1% suggested potential biological processes including musculoskeletal movement, meiotic cell cycle, oocyte maturation and skeletal muscle contraction. Furthermore, pathway analyses suggested potential pathways associated with calving performance traits including tight junction, oxytocin signaling, and MAPK signaling (P < 0.10). The prediction ability of the 1206 significant SNPs was between 78 and 83% of the prediction ability of the BovineSNP50 SNPs for calving performance traits and between 35 and 79% for body conformation traits.

**Conclusions:**

Various SNPs that are significantly associated with calving performance are located within or nearby genes with potential roles in tight junction, oxytocin signaling, and MAPK signaling. Combining the significant SNPs or SNPs within or nearby gene(s) from the HD panel with the BovineSNP50 panel yielded a marginal increase in the accuracy of prediction of genomic estimated breeding values for all traits compared to the use of the BovineSNP50 panel alone.

**Electronic supplementary material:**

The online version of this article (10.1186/s12711-017-0356-8) contains supplementary material, which is available to authorized users.

## Background

The profitability of dairy production depends on the ability of cows for high milk production, good health and fertility. Improving traits such as calving performance and body conformation reduces the culling rate, which in turn affects the profitability of the dairy cattle industry [1–4]. More than 50% of the first lactation heifers may require assistance at calving [5], which increases their culling risk by 18% and reduces their reproductive life [6]. In addition, dams with very difficult calving (score = 4) require eight more days to first service and 28 more days to conceive than those not needing assistance (score = 1) [2]. Furthermore, Dematawewa and Berger [7] reported that cows that experience extreme calving difficulty (score = 5) produce 703 kg of milk, 24 kg of fat and 21 kg of protein less per 305-d of lactation than unassisted cows (score = 1) over multiple parities. Body conformation traits are correlated to economically important traits in dairy cattle such as calving ease [8], longevity [9], lameness [10] and lifetime production efficiency [11]. Improving the accuracy of selection for calving performance and conformation traits would benefit the dairy industry as a whole and have a major impact on the profitability of individual farms and, thus, these traits are included in traditional and genomic breeding programs worldwide [12–15].

Since 2009, significant genetic improvement has been achieved for various dairy cattle traits through the use of high throughput single nucleotide polymorphism (SNP) panels for implementing genomic selection [16]. Increases in the rate of genetic progress were predicted to be as much as 100% [17], and data collected by the Canadian Dairy Network over the last few years support these predictions [18]. The Illumina Bovine SNP50 SNP chip (50 K; Illumina Inc., San Diego, USA) [19] has been used to identify significant regions that are associated with calving [20] and conformation traits [21]. Over the last four years, higher-density SNP chip panels have been developed, including the Illumina BovineHD BeadChip (HD; Illumina Inc., San Diego, USA) and, also, many animals have been sequenced [22]. These HD panels have not had a significant impact on genetic improvement programs, with very minor increases in prediction accuracies over the 50 K panel, either within or across breeds [23, 24]. Nonetheless, HD panels can be used to improve the detection of regions that harbor causal mutations and, in which, genes of interest and new SNPs can be identified.

The objectives of our study were to identify genomic regions associated with 10 calving performance and 28 body conformation traits in Holstein cattle, to retrieve information from associated regions to increase the understanding of the underlying biology of these traits, and to test the predictive ability of these regions in young bulls.

## Methods

### Description of traits

The evaluated traits included 10 calving performance and 28 body conformation traits (Table 1). Calving performance traits were: maternal calving ease at first (heifer; CEh) and later calvings (cow; CEc), maternal calf survival at first (heifer; CSh) and later calvings (cow; CSc), direct calving ease at first (heifer; SCEh) and later calvings (cow; SCEc), direct calf survival at first (heifer; SCSh) and later calvings (cow; SCSc), calving ability index (CA), and daughter calving ability index (DCA). The phenotypes for calving ease were scored as 1 for unassisted, 2 for easy pull, 3 for hard pull and 4 for surgery. Calf survival was scored within 24 h after calving as 0 for dead and 1 for alive. The body conformation traits consisted of 23 linear descriptive traits scored on a 1 to 9 scale and four composite traits which were estimated from linear traits and overall score (www.holstein.ca). A detailed description of the traits and statistical methods for predicting the breeding values of the bulls was previously reported [9, 13, 25–27].

**Table 1:** Heritability and number of records used in the association and validation analyses

Trait	Heritability^a^	Number of records in the training population^b^	Number of records in validation population^c^
*Calving performance*			
Calving ability, CA^d^	0.300	4525	–
Daughter calving ability, DCA^e^	0.060	3892	726
Maternal calving ease at first calving (heifer), CEh	0.121	3908	726
Maternal calf survival at first calving (heifer), CSh	0.056	3873	726
Maternal calving ease at later calvings (cow), CEc	0.084	3879	726
Maternal calf survival at later calvings (cow), CSc	0.023	3530	725
Direct calving ease at first calving (heifer), SCEh	0.017	4523	–
Direct calf survival at first calving (heifer), SCSh	0.004	4361	–
Direct calving ease at later calvings (cow), SCEc	0.016	4516	–
Direct calf survival at later calvings (cow), SCSc	0.003	4315	–
*Rump*			
Rump, RUM	0.233	3573	927
Pin width, PW	0.340	3584	927
Pin setting, PS	0.087	3533	947
Rump angle, RAN	0.365	3573	927
Loin strength, LS	0.251	3576	947
*Body traits*			
Dairy strength, DS	0.359	3584	927
Stature, ST	0.529	3589	927
Height at front end, FE	0.259	3577	947
Chest width, CW	0.218	3582	927
Body depth, BD	0.320	3582	927
Angularity, ANG	0.257	3582	927
*Feet and legs*			
Feet and legs, FL	0.152	3572	927
Foot angle, FAN	0.109	3582	927
Heel depth, HD	0.076	3486	947
Bone quality, BQ	0.300	3584	947
Leg side view, LSV	0.244	3577	927
Set of rear legs, SRL	0.050	3564	947
Leg rear view, LRV	0.125	3555	927
*Udder*			
Mammary system, MS	0.247	3587	927
Udder depth, UD	0.415	3577	927
Udder texture, UT	0.141	3588	947
Median suspensory ligament, MSL	0.140	3581	927
Fore udder attachment, FA	0.282	3587	927
Front teat placement, FTP	0.313	3584	927
Rear attachment height, RAH	0.234	3585	927
Rear attachment width, RAW	0.200	3579	947
Rear teat placement, RTP	0.294	3580	927
Teat length, TL	0.293	3581	927
Overall conformation score, CONF	0.261	3586	927

^a^Heritability = heritability estimates were provided by Canadian Dairy Network

^b^Number of bulls in the training population with de-regressed EBV and used in the association analysis

^c^Number of bulls in the validation population

^d^CA = calving ability index = 0.64 SCEh + 0.16 SCEc + 0.16 SCSh + 0.04 SCSc

^e^DCA = daughter calving ability index = 0.36 CSh + 0.24 CSc + 0.24 CEh + 0.16 CEc

### Genotypes and imputation

Genotypes for HD (774,605 SNPs) and 50 K (40,666 SNPs) SNPs were provided by the Canadian Dairy Network (CND, Guelph, ON, Canada) for 2387 and 11,926 bulls, respectively, both registered and used in North America. The genotyping data was coded as 0, 1, and 2 for BB, AB and AA genotypes, respectively. For purposes of this study, reference population will refer to the animals genotyped with the HD panel used to create the library of haplotypes for the imputation from 50 K to HD genotypes, while target population refers to the bulls genotyped with the 50 K panel and imputed to HD genotypes.

A genotyping quality control (QC) on the HD genotypes removed 39,366 SNPs located on the sex chromosomes, 5427 individual SNPs with a call-rate lower than 90% and 31 SNPs with a heterozygosity that deviates by more than 15% from the expected value under Hardy–Weinberg equilibrium or that are not in Hardy–Weinberg equilibrium (P < 0.0000001). In addition, 3679 SNPs with a high Mendelian error rate (> 0.05) and 6902 SNPs that were assumed to be misplaced and identified based on linkage disequilibrium (LD) decay (see Additional file 1: Figure S1) were excluded. A total of 719,200 SNPs from the HD panel distributed over the 29 *Bos taurus* autosomes (BTA) passed all QC criteria and were used for imputation of the target population. These same QC criteria were applied to the 50 K genotypes and 39,148 SNPs remained for further analyses.

Genotype imputation from the 50 K to HD panel was performed by using the FImpute software and applying a population imputation approach [28]. SNPs with more that 10% of genotypes imputed by random filling based on the allele frequencies in the reference population had these imputed genotypes set to missing using the *gp_thresh* option in the FImpute software and, in a further QC step, these SNPs were excluded from further analyses.

To estimate the accuracy of imputation, 387 animals were randomly selected from the reference population of animals with HD genotypes and their genotypes were masked down to the 50 K panel (39,148 SNPs). Then, the genotypes for these animals were imputed to HD by using the remaining 2000 animals in the reference population. The squared correlation (r^2^) between imputed genotypes and true genotypes was used as a measure of imputation accuracy [29]. For the genome-wide association studies (GWAS) and genomic prediction of breeding values, imputation was carried out using all animals in the reference population (n = 2387).

After imputation, an additional QC was performed on the real and imputed genotypes. This step excluded 117,482 SNPs that had a minor allelic frequency (MAF) lower than 0.01 and one SNP for which heterozygosity deviated by more than 15% from the expected value under Hardy–Weinberg equilibrium. There were no animals and no individual SNPs with a call rate lower than 90%. A total of 601,717 SNPs distributed over the 29 bovine autosomes passed the QC procedure (see Additional file 2: Figure S2). To examine population stratification among the bulls in the training population, the HD genotypes were used to perform a multidimensional scaling analysis based on identical-by-state (IBS) pairwise distances, as implemented in PLINK software [30].

### De-regressed estimated breeding values for the training population

Domestic official evaluations of 10 calving performance and 28 body conformation traits, and their associated reliabilities from the April 2009 genetic evaluation, were provided by the Canadian Dairy Network (CDN; www.cdn.ca) for 4841 progeny-tested Holstein bulls born between 1956 and 2007. These progeny-tested Holstein bulls had either a real HD (n = 287) or an imputed HD (n = 4554) genotype. The de-regressed estimated breeding values of bulls were computed by CDN following VanRaden et al. [31]:

1 $${\text{De}} - {\text{EBV}} = {\text{PA}} + \left( {{\text{EBV}} - {\text{PA}}} \right)/{\text{Rel}}_{{{\text{De}} - {\text{EBV}}}} ,$$

where ${\text{De}} - {\text{EBV}}$ is the bull’s de-regressed estimated breeding value for the trait of interest, ${\text{PA}}$ is the parent average and ${\text{Rel}}_{{{\text{De}} - {\text{EBV}}}}$ is the reliability of the bull’s EBV adjusted to remove the contribution of ${\text{PA}}$. For purposes of this study, training population refers to the population of bulls that had both pseudo-phenotypes (${\text{De}} - {\text{EBV}}$) and genotypes used for the estimation of the effects of SNPs, while validation population refers to the animals used for the validation of the effects of SNPs estimated by using the training population.

Pedigree data for 23,287 Holsteins individuals were obtained from CDN. The level of pedigree completeness of the training population was assessed using the pedigree completeness index (${\text{PCI}}$) proposed by MacCluer et al. [32] and implemented in the CFC package [33]. The ${\text{PCI}}$ was calculated as:

2 $${\text{PCI}} = \frac{{K_{dam} K_{sire} }}{{K_{dam} + K_{sire} }},$$

where the *K*_*dam*_ and *K*_*sire*_ are the indexes for dams and the sires, respectively, given by:

$$K = \frac{1}{g}\mathop \sum \limits_{i = 1}^{g} a_{i}$$

where *a*_*i*_ is the proportion of known ancestors in generation *i* for either the dams or the sires, and *g* is the number of generations back (5 in our study).

### Genome-wide association analyses

The GWAS were performed using an univariate single-SNP mixed linear model implemented in the snp1101 software [34]. The mixed model equations are described as:

3 $$\left[ {\begin{array}{*{20}c} {{\bf{1}} '{\mathbf{R}}^{ - 1} {\bf{1}}} & {{\bf{1}} '{\mathbf{R}}^{ - 1} {\mathbf{X}}} & {{\bf{1}} '{\mathbf{R}}^{ - 1} {\mathbf{Z}}} \\ {{\mathbf{X}} '{\mathbf{R}}^{ - 1} {\bf{1}}} & {{\mathbf{X}} '{\mathbf{R}}^{ - 1} {\mathbf{X}}} & {{\mathbf{X}} '{\mathbf{R}}^{ - 1} {\mathbf{Z}}} \\ {{\mathbf{Z}} '{\mathbf{R}}^{ - 1} {\bf{1}}} & {{\mathbf{Z}} '{\mathbf{R}}^{ - 1} {\mathbf{X}}} & {{\mathbf{Z^{\prime}R}}^{ - 1} {\mathbf{Z}} + {\mathbf{G}}^{ - 1} *\frac{{\upsigma_{\text{e}}^{2} }}{{\upsigma_{\text{g}}^{2} }}} \\ \end{array} } \right] \times \left[ {\begin{array}{*{20}c} {{\hat{\upmu }}} \\ {{\hat{\upbeta }}} \\ {{\hat{\mathbf{u}}}} \\ \end{array} } \right] = \left[ {\begin{array}{*{20}c} {{\bf{1}} '{\mathbf{R}}^{ - 1} {\mathbf{Y}}} \\ {{\mathbf{X}} '{\mathbf{R}}^{ - 1} {\mathbf{Y}}} \\ {{\mathbf{Z}} '{\mathbf{R}}^{ - 1} {\mathbf{Y}}} \\ \end{array} } \right],$$

where ${\mathbf{Y}}$ is the vector of the ${\text{De}} - {\text{EBV}}$ for each trait; **1** is a vector of ones, ${\mathbf{X}}$ is the vector of the animals’ genotypes for a given SNP, coded as 0, 1 and 2; ${\mathbf{Z}}$ is the design matrix that assigns animals to ${\text{De}} - {\text{EBV}}$; ${\hat{\upmu }}$ is the overall mean, ${\hat{\upbeta }}$ is the linear regression coefficient (allele substitution effect) of the examined SNP; ${\hat{\mathbf{u}}}$ is a vector of direct genomic values (DGV). Assumptions for the model were: ${\mathbf{u}}\sim N\left( {0, {\mathbf{G}}\upsigma_{\text{g}}^{2} } \right)$ where ${\mathbf{G}}$ is the genomic relationship matrix, ${\mathbf{R}}$ is a diagonal matrix containing weights for the residual variance based on the reliabilities of the ${\text{De}} - {\text{EBV}}$. The diagonal elements of ${\mathbf{R}}$ are given by $\left( {\frac{1}{{1 - \frac{1}{{{\text{Rel}}_{\text{i}} }}}}} \right)$ [35], where ${\text{Rel}}_{\text{i}}$ is the reliability of the ${\text{i}}$th ${\text{De}} - {\text{EBV}}$; $\upsigma_{\text{g}}^{2}$ is the additive genetic variance; and, $\upsigma_{\text{e}}^{2 }$ is the residual variance. The values of $\upsigma_{\text{g}}^{2}$ and $\upsigma_{\text{e}}^{2}$ were estimated using the AI-REML algorithm implemented in the snp1101 software [34]. Allele frequencies used to calculate ${\mathbf{G}}$ were estimated from the observed genotype data. The ${\mathbf{G}}$ matrix was calculated as:

4 $${\mathbf{G}} = {\mathbf{CC^{\prime}}}/2\sum {\text{P}}_{\text{i}} (1 - {\text{P}}_{\text{i}} ) ,$$

where ${\mathbf{C}}$ is the genotypic coefficient matrix with dimensions equal to the number of genotyped animals by the number of SNPs and ${\text{P}}_{\text{i}}$ is the allele frequency of the ${\text{i}}$th SNP. The relationship between individuals ${\text{j}}$ and ${\text{k}}$ is ${\text{G}}_{\text{jk}}$ divided by the square root of the diagonal values of ${\text{j}}$ (${\text{G}}_{\text{jj}}$) and ${\text{k}}$ (${\text{G}}_{\text{kk}}$) individuals [35].

The significance of the effects of SNPs was determined by using a genome-wise false discovery rate (FDR) of 5% [36]. The more lenient FDR threshold of 5% was used to increase the power to detect SNPs with small effects since traits of interest may be controlled by many QTL with a small effect [37]. The genomic inflation factor (λ) and quantile–quantile (Q–Q) plots were applied to assess the inflation of the test statistics due to population stratification by comparing the genome-wide distribution of the test statistic (− log10 of *P*-values) with the expected Chi squared distribution. The significant SNPs at a genome-wise FDR of 5% were aligned to the publicly available QTL in the Animal QTLdb (Release 26, access date: April 27th, 2015) [38].

### In-silico functional analyses

Candidate SNP enrichment analyses were performed on the GWAS results using the SNP2GO R package [39] for gene ontology (GO). To infer overrepresentation of candidate SNPs (i.e., the list of significant SNPs at a *P* value < 0.05), the *Bos taurus* annotations from Ensembl version 75 for the UMD 3.1 assembly, the associated GO terms, and the list of candidate and non-candidate (i.e., non-significant) SNPs were used as input for SNP2GO. In the SNP2GO analyses, significant SNPs were assigned to plus or minus 50 nucleotides up- and down-stream of the corresponding genes. To account for multiple testing in the SNP enrichment analysis, a FDR of 1% was applied.

Pathway analyses were performed on the list of genes obtained by mapping the significant SNPs from the GWAS to their corresponding genes or nearby genes at a distance of 10 kbp using NGS-SNP [40], the bovine genome assembly UMD3.1 and the Ensembl database 75_31. The 10 kbp distance was used to exploit the expected LD (r^2^) between pairs of syntenic SNPs, which had an average r^2^ of 0.58 [41]. The pathway analyses were performed using the web-based Database for Annotation, Visualization, and Integrated Discovery (DAVID) version 6.8 [42].

### Validation study

We performed a forward validation study, in which the Canadian domestic estimated breeding values from the April 2009 genetic evaluation were used to estimate SNP associations and direct genomic breeding values (DGV). Unproven young bulls in the April 2009 genetic evaluation with estimated breeding values in December 2014 were considered for the validation population (Table 1). The DGV for the validation group were estimated by using the genomic best linear unbiased prediction method (GBLUP) [35, 43, 44] implemented in the gebv software [45]. In this method, the DGV were estimated using the following mixed model equations:

5 $$\left[ {\begin{array}{*{20}c} {{\bf{1}}'{\mathbf{R}}^{ - 1} {\bf{1}}} \\ {{\mathbf{Z^{\prime}R}}^{ - 1} {\bf{1}}} \\ \end{array} \begin{array}{*{20}c} {{\bf{1}} '{\mathbf{R}}^{ - 1} {\mathbf{Z}}} \\ { {\mathbf{Z}}^{ '} {\mathbf{R}}^{ - 1} {\mathbf{Z}} + {\mathbf{G}}_{\text{w}}^{ - 1} \frac{{\upsigma_{\text{e}}^{2} }}{{\upsigma_{\text{g}}^{2} }}} \\ \end{array} } \right]\left[ {\begin{array}{*{20}c} {{\hat{\upmu }}} \\ {{\hat{\mathbf{u}}}} \\ \end{array} } \right] = \left[ {\begin{array}{*{20}c} {{\bf{1}} '{\mathbf{R}}^{ - 1} {\mathbf{Y}}} \\ {{\mathbf{Z}} '{\mathbf{R}}^{ - 1} {\mathbf{Y}}} \\ \end{array} } \right],$$

where ${\mathbf{Y}}$ is the vector of ${\text{De}} - {\text{EBV}}$ from the April 2009 genetic evaluation for the trait of interest for genotyped bulls, ${\hat{\upmu }}$ is the overall mean, **1** is a vector of ones, ${\mathbf{Z}}$ is the design matrix that assigns animals to ${\text{De}} - {\text{EBV}}$, ${\hat{\mathbf{u}}}$ is a vector of DGV. Assumptions for this model are: ${\mathbf{u}}{\sim}N\left( {0, {\mathbf{G}}_{\text{w}} {{\upsigma }}_{\text{g}}^{2} } \right)$, where ${\mathbf{G}}_{\text{w}} = {\text{w}}^{ *} {\mathbf{G}} + \left( {1 - {\text{w}}} \right)^{{*}} {\mathbf{A}}$ and $\upsigma_{\text{g}}^{2}$ is the additive genetic variance; ${\mathbf{G}}$ is the genomic relationship matrix [35], ${\mathbf{A}}$ is the numerator relationship matrix and ${\text{w}}$ is the weight put on ${\mathbf{G}}$ (0.8). As in Model (3), ${\mathbf{R}}$ is a diagonal matrix with elements defined as R_ii_ = $\left( {\frac{1}{{1 - \frac{1}{{{\text{Rel}}_{\text{i}} }}}}} \right)$ where ${\text{Rel}}_{\text{i}}$ is the reliability of the ${\text{i}}$th ${\text{De}} - {\text{EBV}}$; $\upsigma_{\text{e}}^{2}$ is the error variance.

Different scenarios for the validation analyses were tested depending on the number of SNPs used in the prediction. Scenario 1 used all the SNPs in the 50 K panel that passed the QC (38,724 SNPs), Scenario 2 used all the SNPs in the HD panel that passed the QC (607,794 SNPs), Scenario 3 used only a set of significant (genome-wise FDR of 5%) SNPs for all calving performance and conformation traits, Scenario 4 combined SNPs from the 50 K panel and the significant SNPs not in the 50 K panel, and Scenario 5 combined a set of significant SNPs for calving performance and conformation traits within or nearby the gene (± 100 kbp around the gene) plus the 50 K panel. The accuracy of genomic breeding values was calculated as the squared correlation (r^2^) between DGV and official genetic evaluations from December 2014 for the validation group.

## Results and discussion

### Population structure

The results from the multidimensional scaling analysis showed no divergent clusters within this population (see Additional file 3: Figure S3). In addition, the pedigree analysis indicated that the overall ${\text{PCI}}$ considering five generations back was equal to 0.62. Although the ${\text{PCI}}$ for the reference population was high, modeling the relationship between animals using the pedigree information may not completely account for population structure because of missing pedigree records. The current Holstein cattle population was characterized by a high rate of inbreeding, genetic diversity loss and small effective population size [46, 47] and would probably have cryptic relationships that are not described by the available pedigree. Thus, the population structure and cryptic relatedness not accounted for by an incomplete pedigree should be accounted for in GWAS to avoid spurious associations [48].

### Accuracy of genome-wide imputation

After applying QC criteria, 719,200 SNPs distributed over the 29 bovine autosomes in the reference population (2387 individuals) were used for genome-wide imputation of the target population (11,926 animals) for which 39,148 SNPs were available. The squared correlation (r^2^) between imputed genotypes and true genotypes of the target population was estimated at 99.29%, which indicated that the imputation was highly accurate. The allelic r^2^ was used as a measure of imputation accuracy because it adequately reflects the imputation accuracy of SNPs with a low MAF [29]. In this study, the accuracy of imputation was high for several reasons. First, the reference population with HD genotypes was relatively large (n = 2387) compared with other studies that reported high imputation accuracies by using the FImpute software and with less individuals in the reference group (e.g., 1733) than those used in our study [28]. In addition, the accuracy of imputation from 50 K to HD using FImpute was also high (99.96%) in a Holstein reference population of limited size (n = 1406) [24]. Second, the reference population in our study shares relatively long-range haplotypes [28] with high phasing accuracy which also contributes to higher imputation accuracies [49]. Third, this reference population is also closely related to the target population which helped increase the accuracy of imputation, even for SNPs with a low MAF (≤ 0.05) [28]. Furthermore, an internal score based on the accuracy of imputation for each SNP allele was used to set less accurate imputed alleles to missing and the SNPs with a call rate lower than 90% were excluded in the QC before GWAS. In addition, SNPs with a MAF lower than 1% were initially excluded, which resulted in more accurate genotypes for the GWAS. The use of imputation increased both the numbers of SNPs and animals included in the association analysis, which increased the power of QTL detection [50] and the accuracy of genomic prediction of breeding values [51, 52]. Combining SNP panels with different densities and using imputed genotypes in QTL mapping is a common approach in human GWAS [53] and in dairy cattle genomic evaluations [24, 54].

### Genome-wide association for calving performance traits

In the GWAS, family and cryptic stratifications were accounted for by incorporating the full genomic covariance among individuals. The genomic inflation factor was used to assess bias in the test statistics. The average genomic inflation factor was equal to 0.98 and ranged from 0.93 for SCEc to 1.00 for heel depth (see Additional file 4: Figure S4), which suggests that any potential bias due to population stratification was addressed [55–57]. Furthermore, accounting for the reliability of the de-regressed EBV allowed the use of more records in the estimation step by including de-regressed EBV with lower reliability, which subsequently improved the accuracy of GWAS [50, 52].

**Fig. 1:**
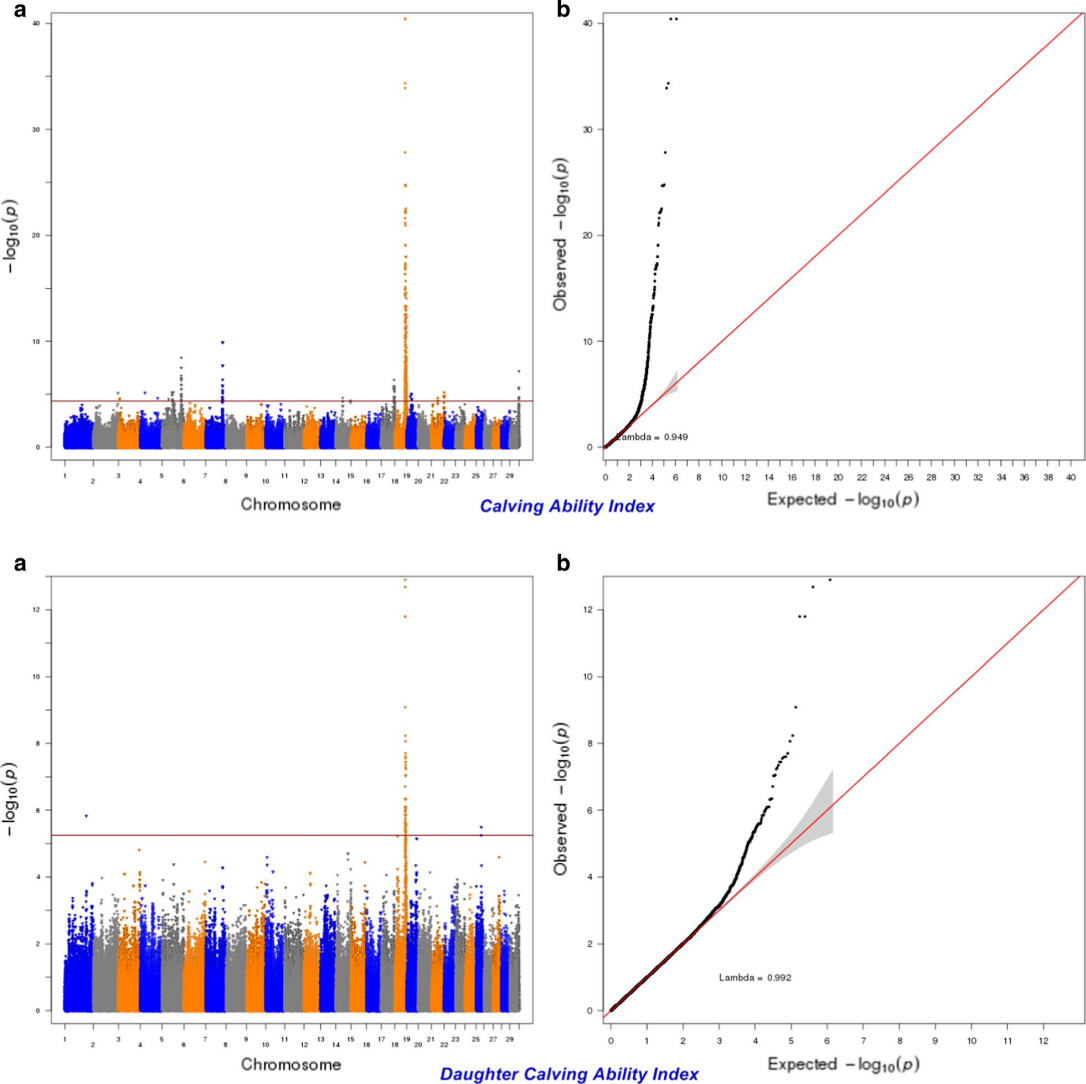
Manhattan (**a**) and Q–Q (**b**) plots from GWAS for calving ability index and daughter calving ability index using Illumina BovineHD BeadChip in Holstein cattle. **a** Manhattan plot in which the genomic coordinates of SNPs are displayed along the horizontal axis, the negative logarithm of the association P-value for each SNP is displayed on the vertical axis, and the dark red line is the significance threshold line at the genome-wise false discovery rate of 5%. **b** Quantile–quantile (Q–Q) plot showing the late separation between observed and expected values. Genomic inflation factor is around 1 indicating that there is no population stratification

**Fig. 2:**
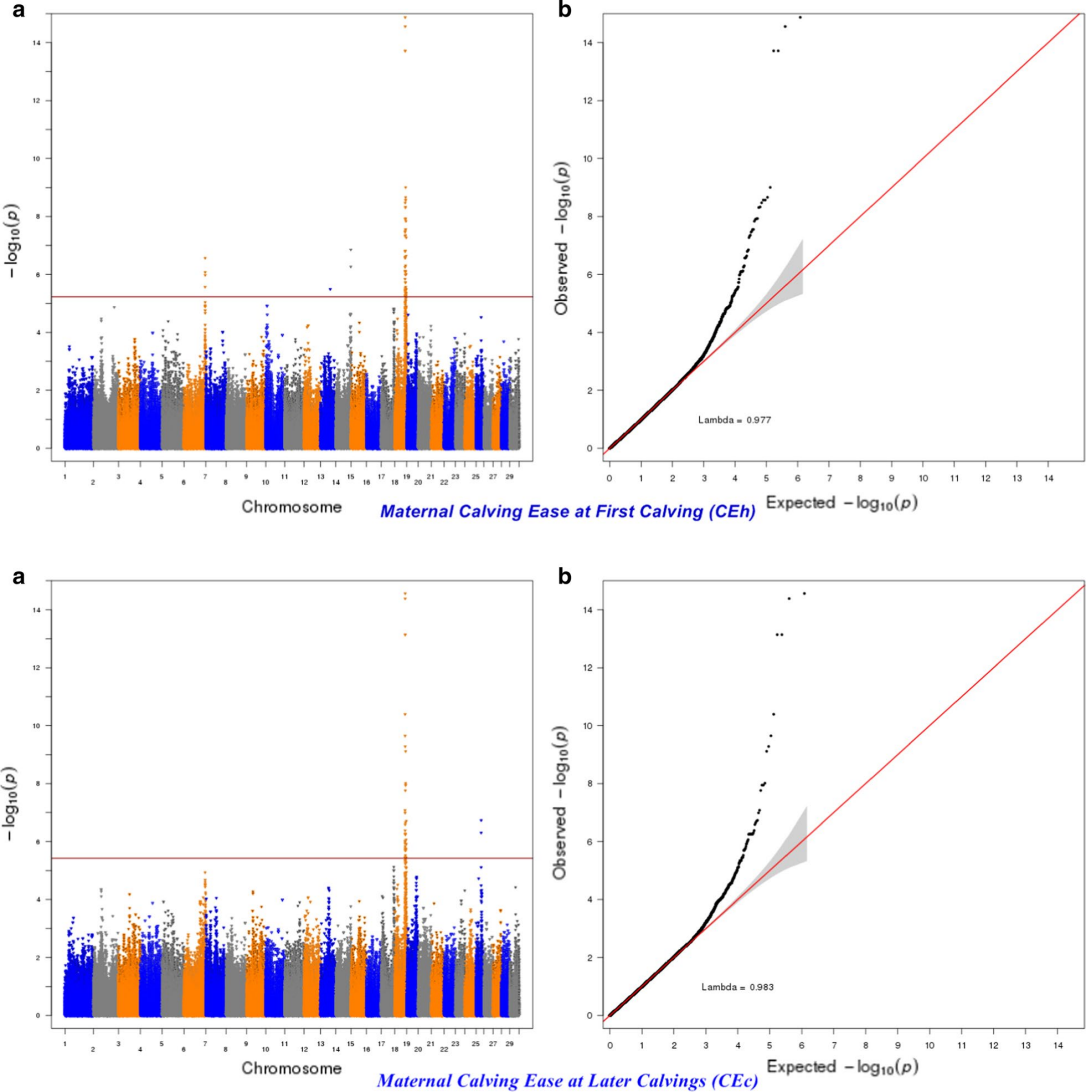
Manhattan (**a**) and Q–Q (**b**) plots from GWAS for maternal calving ease at first calving and maternal calving ease at later calvings using Illumina BovineHD BeadChip in Holstein cattle. **a** Manhattan plot in which the genomic coordinates of SNPs are displayed along the horizontal axis, the negative logarithm of the association P-value for each SNP is displayed on the vertical axis, and the dark red line is the significance threshold line at the genome-wise false discovery rate of 5%. **b** Quantile–quantile (Q–Q) plot showing the late separation between observed and expected values. Genomic inflation factor is around 1 indicating that there is no population stratification

**Fig. 3:**
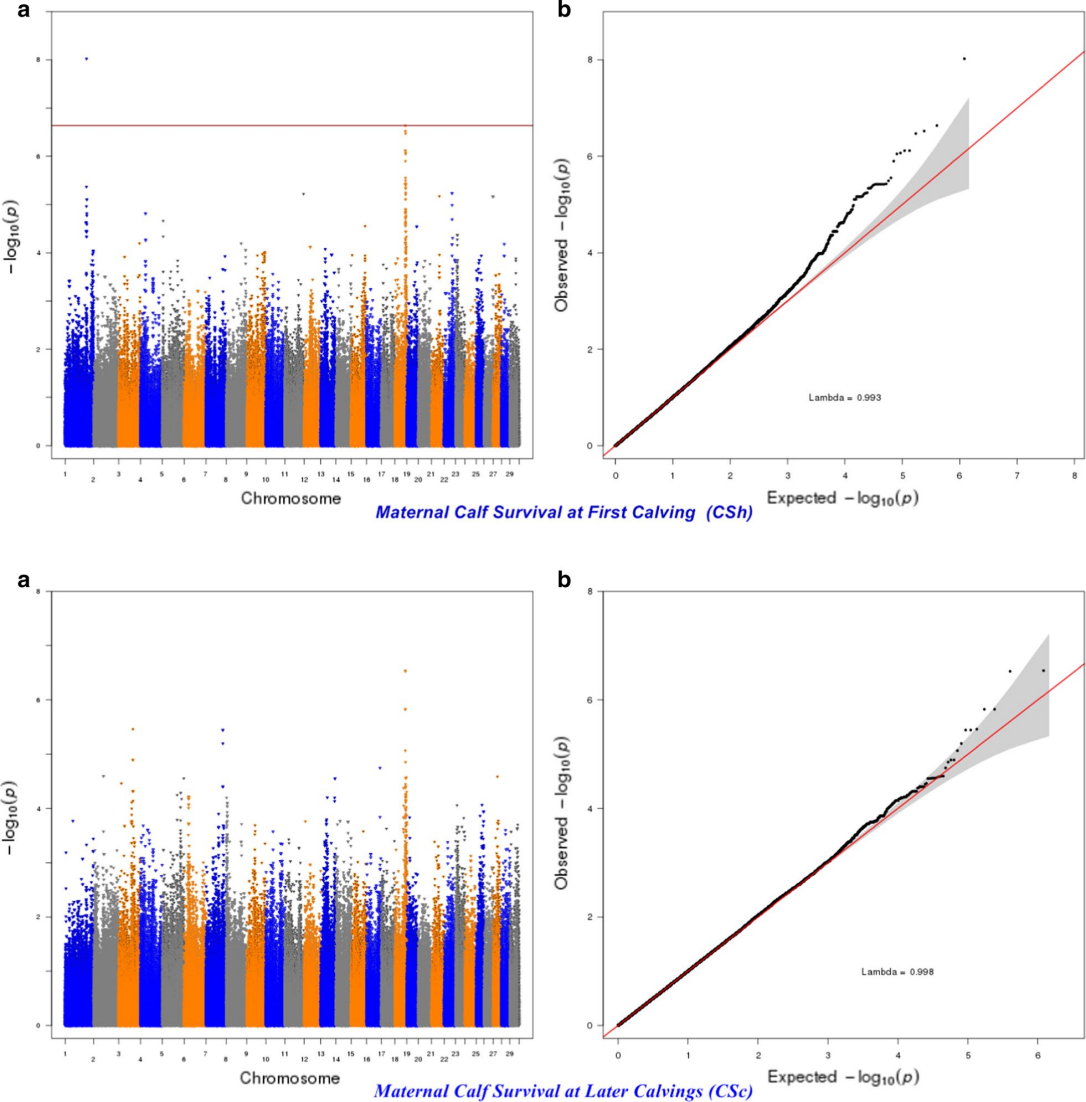
Manhattan (**a**) and Q–Q (**b**) plots from GWAS for maternal calf survival at first calving and maternal calf survival at later calvings using Illumina BovineHD BeadChip in Holstein cattle. **a** Manhattan plot in which the genomic coordinates of SNPs are displayed along the horizontal axis, the negative logarithm of the association P-value for each SNP is displayed on the vertical axis, and the dark red line is the significance threshold line at the genome-wise false discovery rate of 5%. **b** Quantile–quantile (Q–Q) plot showing the late separation between observed and expected values. Genomic inflation factor is around 1 indicating that there is no population stratification

**Fig. 4:**
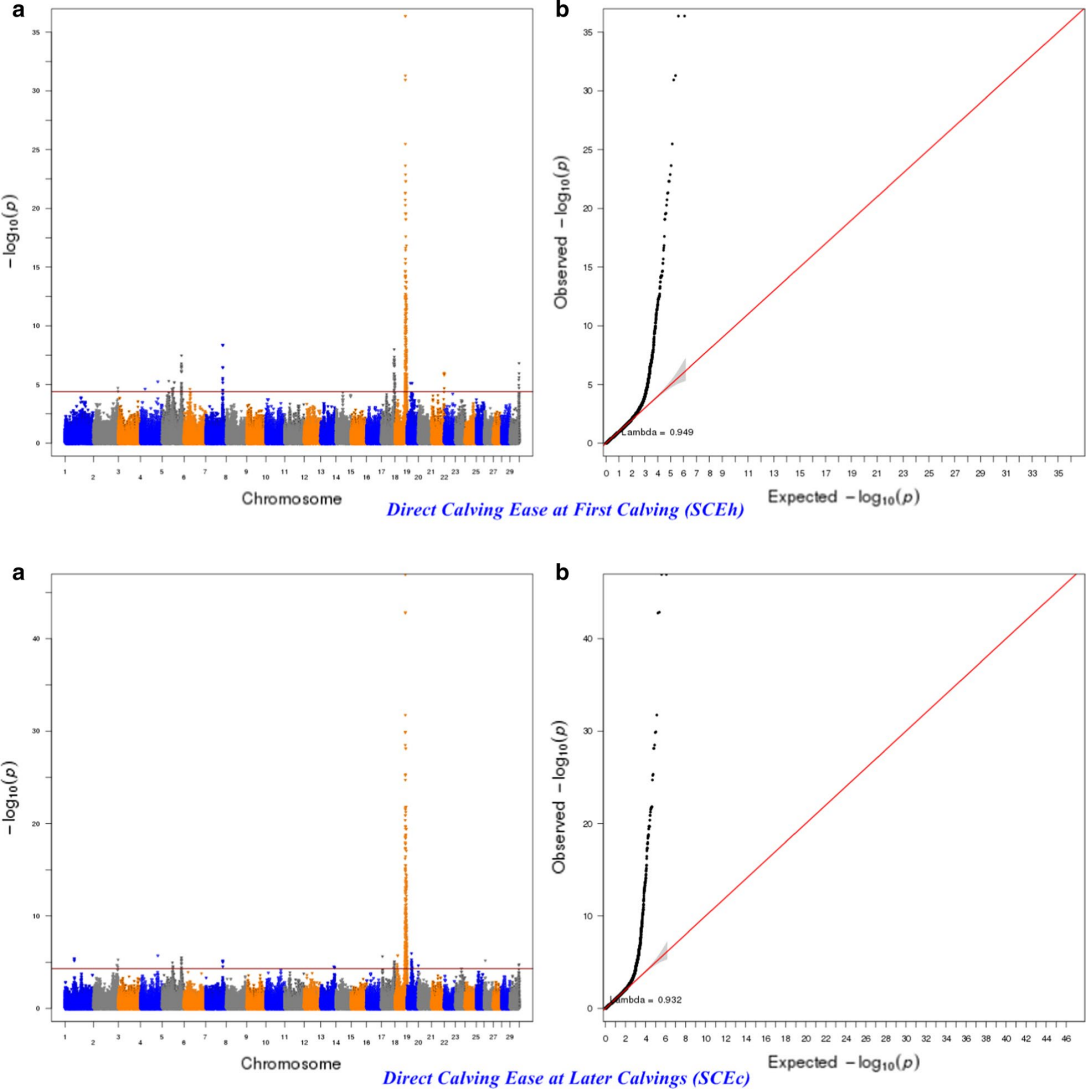
Manhattan (**a**) and Q–Q (**b**) plots from GWAS for direct calving ease at first calving and direct calving ease at later calvings using Illumina BovineHD BeadChip in Holstein cattle. **a** Manhattan plot in which the genomic coordinates of SNPs are displayed along the horizontal axis, the negative logarithm of the association P-value for each SNP is displayed on the vertical axis, and the dark red line is the significance threshold line at the genome-wise false discovery rate of 5%. **b** Quantile–quantile (Q–Q) plot showing the late separation between observed and expected values. Genomic inflation factor is around 1 indicating that there is no population stratification

**Fig. 5:**
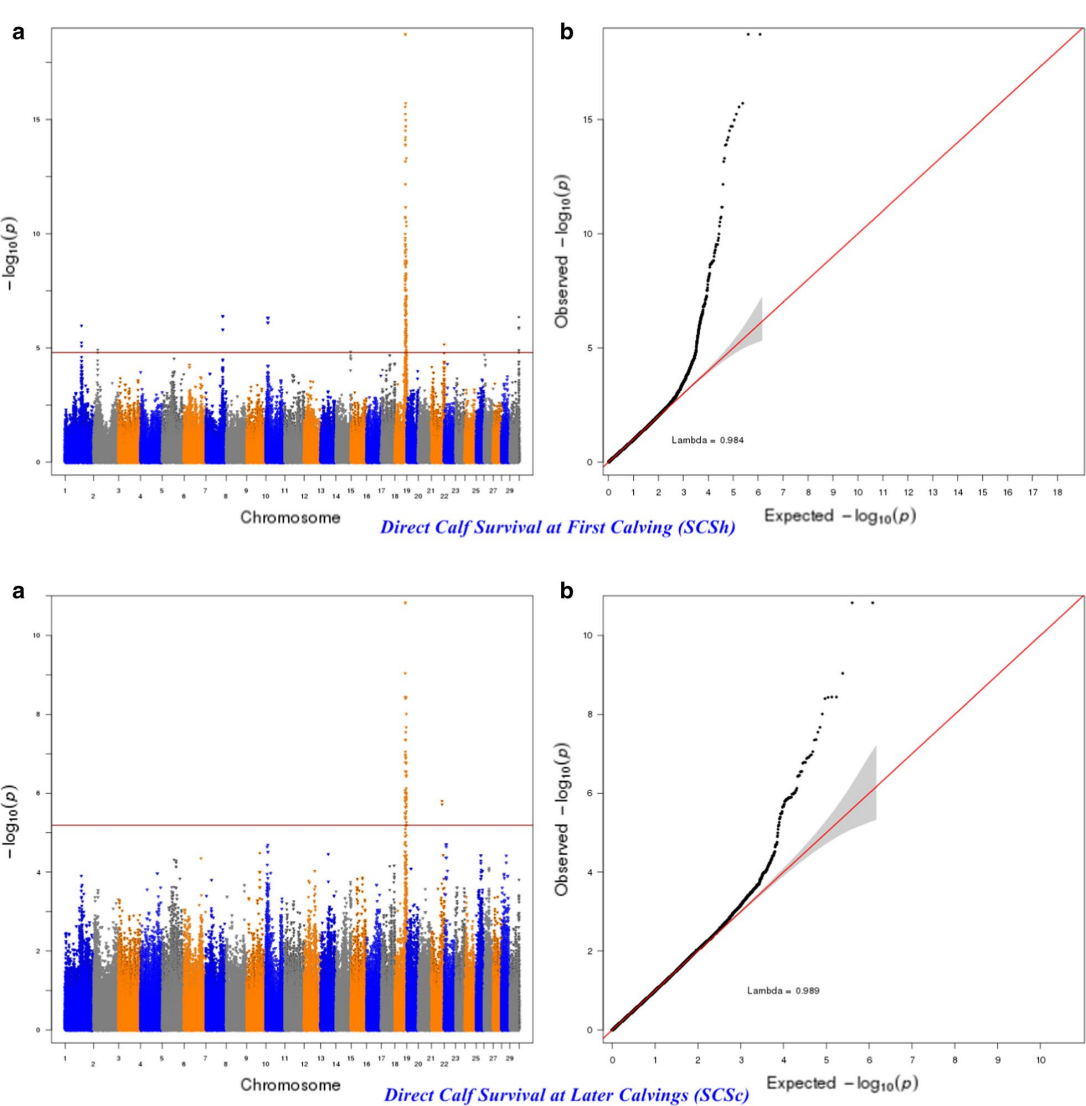
Manhattan (**a**) and Q–Q (**b**) plots from GWAS for direct calf survival at first calving and direct calf survival at later calvings using Illumina BovineHD BeadChip in Holstein cattle. **a** Manhattan plot in which the genomic coordinates of SNPs are displayed along the horizontal axis, the negative logarithm of the association P-value for each SNP is displayed on the vertical axis, and the dark red line is the significance threshold line at the genome-wise false discovery rate of 5%. **b** Quantile–quantile (Q–Q) plot showing the late separation between observed and expected values. Genomic inflation factor is around 1 indicating that there is no population stratification

**Fig. 6:**
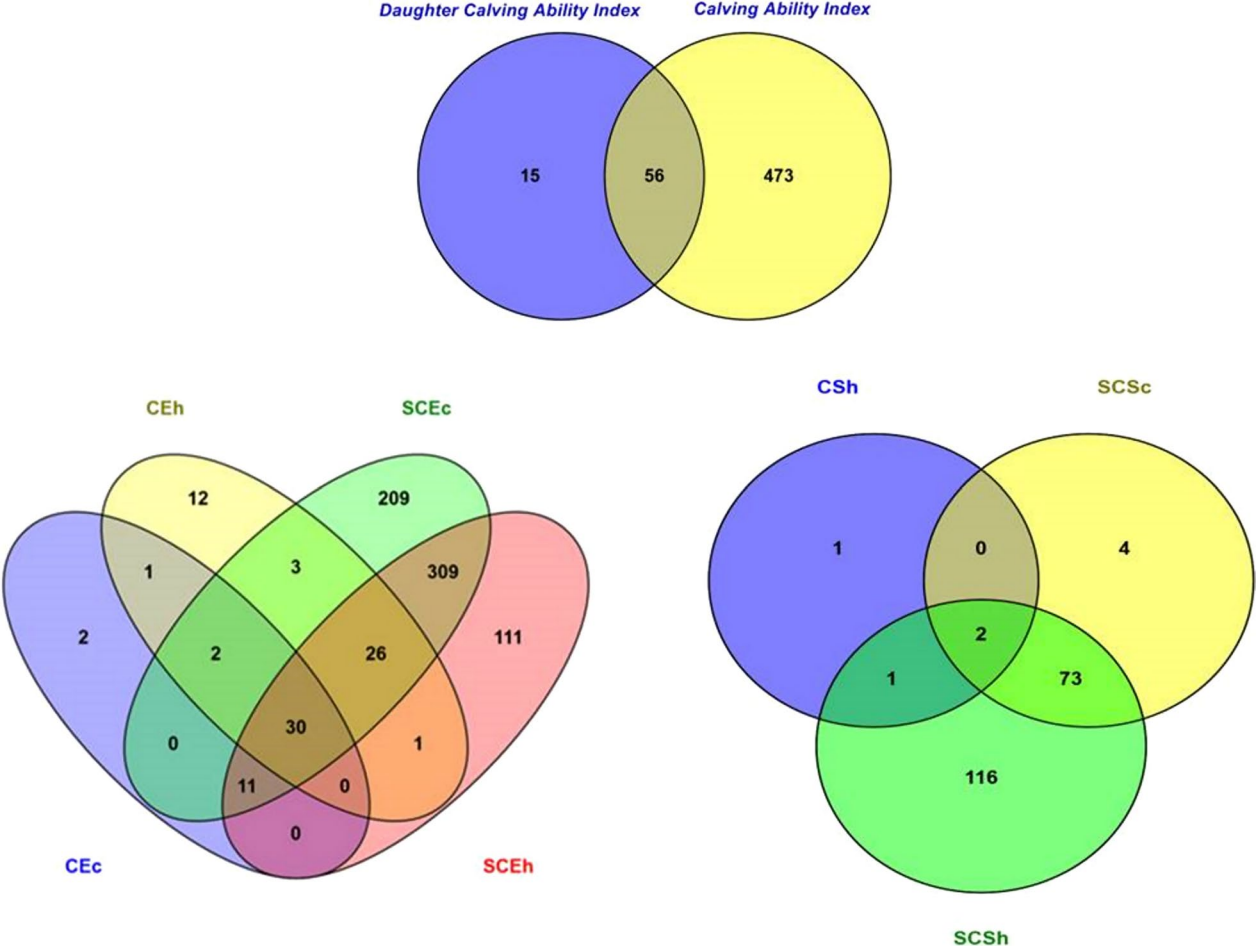
Number of significant (at the genome-wise false discovery rate of 5%) SNPs that have a pleiotropy effect on calving performance using the Illumina BovineHD BeadChip in Holstein cattle. Calving performance traits are calving ability (CA); daughter calving ability (DCA); maternal calving ease at first calving (heifer; CEh); maternal calf survival at first calving (heifer; CSh); maternal calving ease at later calvings (cow; CEc); maternal calf survival at later calvings (cow; CSc); direct calving ease at first calving (heifer; SCEh); direct calf survival (heifer; SCSh); direct calving ease at later calvings (cow; SCEc); sire calf survival (cow; SCSc)

For calving performance, 782 SNPs significant at a genome-wide FDR of 5% were identified. Most of these significant SNPs were on BTA18 (71.87%), 17 (7.42%), 5 (6.78%), 7 (2.43%), 19 (1.92%), 21 (1.66%), 29 (1.66%), 1 (1.02%) and 10 (1.02%). The significant SNPs were mapped to 675 genes, among which 142 included at least one significant SNP and 532 were nearby (100 kbp) a SNP. A strong peak on BTA18 that affected maternal and direct calving ease and calf survival in the first and later calvings was identified (Figs. 1, 2, 3, 4, 5). Previously, Sahana et al. [58] and Hoglund et al. [59] reported QTL on BTA18 that were associated with direct and maternal calving ease, calf size, stillbirth and birth index in Danish and Swedish Holstein cattle. In addition, Saatchi et al. [60] reported a QTL on BTA18 (at 54.37 Mb) that was associated with maternal calving ease in Simmental beef cattle. Also, the significant associations identified for most calving performance traits on BTA5, 6, 7, 9, 13, 17, 18, 19, 20 and 23 are in agreement with previous studies [58–60]. The significant associations were located within the confidence interval of previously detected QTL (see Additional file 5: Table S1). Thirty significant (FDR of 5%) SNPs were common between maternal and direct calving performance traits (Fig. 6).

Several genes that contain significant SNPs for calving performance traits were identified (Table 2) and are involved in biological processes such as lipid metabolism, immunity, reproduction and anatomical structure development. For example, *kallikrein*-*related peptidase 14* (*KLK14*) is involved in mating and reproduction processes in a multicellular organism [61, 62]. *KLK14* was reported to play an important role in the synergistic effects between estrogens and androgens [61] and at the onset of parturition [62]. Another important gene was *killer cell immunoglobulin like receptor, two Ig domains and long cytoplasmic tail 5A* (*KIR2DL5A*), which is attributed to Graft-versus-host disease and natural killer cell mediated cytotoxicity [63]. The *membrane bound O*-*acyltransferase domain containing 7* (*MBOAT7*) gene is involved in the metabolism of lipids and lipoproteins, including the pathway of glycerophospholipids metabolism, which is linked to energy balance metabolites including non-esterified fatty acids (NEFA), beta-hydroxybutyrate (BHBA) and glucose in animals [64]. It is well documented that the period around calving is associated with several biological processes related to fat, protein, glucose and mineral metabolism as well as complex hormonal changes [65]. Monitoring and nutritional modeling of the animal’s energy balance to prevent negative energy balance during the transition period of dairy cows is an important management practice that can reduce the incidence of metabolic and infectious diseases [66]. Energy balance could be associated with the length of the dry period and subsequently with calving difficulty which is generally common under a short (< 60 days) compared to a long dry period [67].

**Table 2:** The 10 most significant SNPs associated with each calving performance trait at the genome-wise false discovery rate of 5%

Trait^a^	RefSNP (rs)^b^	c	Pos. (bp)	MAF	Estimated effect	SE	P-value	Location^d^	Gene name
CA	rs109478645	18	57589121	0.12	− 5.55	0.39	3.69E−41	5484 L	*LOC100138951*
CA	rs136283363	18	57548213	0.12	5.55	0.39	3.69E−41	5497L	*LOC615876*
CA	rs110801791	18	57516245	0.13	− 4.86	0.38	4.51E−35	1252 R	*CTU1*
CA	rs135253383	18	57520290	0.13	4.83	0.38	1.27E−34	Within	*CTU1*
CA	rs136514789	18	57486184	0.16	3.92	0.34	1.50E−28	1330 L	*LOC540297*
CA	rs109882115	18	58067310	0.28	− 3.15	0.29	1.71E−25	Within	*ENSBTAG00000039491*
CA	rs132734257	18	57511637	0.19	− 3.54	0.33	2.04E−25	5860 R	*CTU1*
CA	rs135508298	18	59602905	0.21	3.52	0.33	2.12E−25	75902 L	*ENSBTAG00000048191*
CA	rs134539659	18	59612379	0.21	− 3.52	0.33	2.12E−25		
CA	rs109540436	18	59890631	0.20	3.37	0.33	2.98E−23	3982 R	*LOC100300095*
CEc	rs110801791	18	57516245	0.13	− 3.93	0.49	2.72E−15	1252 R	*CTU1*
CEc	rs135253383	18	57520290	0.13	3.90	0.49	4.08E−15	Within	*CTU1*
CEc	rs109478645	18	57589121	0.12	− 3.86	0.51	7.21E−14	5484 L	*LOC100138951*
CEc	rs136283363	18	57548213	0.12	3.86	0.51	7.21E−14	5497L	*LOC615876*
CEc	rs137554975	18	56944300	0.15	− 2.98	0.45	4.04E−11	Within	*MYH14*
CEc	rs136113894	18	56959011	0.16	− 2.82	0.44	2.25E−10	Within	*KCNC3*
CEc	rs136514789	18	57486184	0.16	2.79	0.44	5.19E−10	1330 L	*LOC540297*
CEc	rs133544315	18	60019327	0.21	2.61	0.42	7.62E−10	47852 L	*ZNF836*
CEc	rs109540436	18	59890631	0.20	2.45	0.42	9.69E−09	3982 R	*LOC100300095*
CEc	rs135033267	18	60043680	0.20	− 2.45	0.43	1.13E−08	25523 R	*ENSBTAG00000011844*
CEh	rs110801791	18	57516245	0.13	− 3.73	0.46	1.33E−15	1252 R	*CTU1*
CEh	rs135253383	18	57520290	0.13	3.69	0.46	2.77E−15	Within	*CTU1*
CEh	rs109478645	18	57589121	0.12	− 3.73	0.48	1.92E−14	5484 L	*LOC100138951*
CEh	rs136283363	18	57548213	0.12	3.73	0.48	1.92E−14	5497L	*LOC615876*
CEh	rs133544315	18	60019327	0.21	2.45	0.40	9.84E−10	47852 L	*ZNF836*
CEh	rs109540436	18	59890631	0.20	2.41	0.40	2.17E−09	3982 R	*LOC100300095*
CEh	rs135033267	18	60043680	0.20	− 2.41	0.40	2.69E−09	25523 R	*ENSBTAG00000011844*
CEh	rs137436636	18	60006650	0.20	2.41	0.40	2.69E−09	35175 L	*ZNF836*
CEh	rs136514789	18	57486184	0.16	2.49	0.42	3.36E−09	1330 L	*LOC540297*
CEh	rs133144614	18	59242260	0.41	− 1.99	0.34	4.68E−09	13197 L	*ZNF845*
CSh	rs43264905	1	118222449	0.04	4.91	0.85	9.51E−09	15187 L	*ENSBTAG00000038069*
DCA	rs110801791	18	57516245	0.13	− 4.27	0.57	1.26E−13	1252 R	*CTU1*
DCA	rs135253383	18	57520290	0.13	4.23	0.57	2.06E−13	Within	*CTU1*
DCA	rs109478645	18	57589121	0.12	− 4.25	0.59	1.59E−12	5484 L	*LOC100138951*
DCA	rs136283363	18	57548213	0.12	4.25	0.59	1.59E−12	5497L	*LOC615876*
DCA	rs136514789	18	57486184	0.16	3.18	0.51	8.24E−10	1330 L	*LOC540297*
DCA	rs41601357	18	58800104	0.49	2.33	0.40	5.86E−09	7804 L	*LOC101904049*
DCA	rs133144614	18	59242260	0.41	− 2.41	0.42	8.66E−09	13197 L	*ZNF845*
DCA	rs109011289	18	59058582	0.48	− 2.27	0.40	1.99E−08	7587 L	*LOC101904371*
DCA	rs41582522	18	58666276	0.41	− 2.33	0.41	2.47E−08		
DCA	rs133544315	18	60019327	0.21	2.76	0.49	2.55E−08	47852 L	*ZNF836*
SCEc	rs109478645	18	57589121	0.12	− 6.19	0.40	1.07E−47	5484 L	*LOC100138951*
SCEc	rs136283363	18	57548213	0.12	6.19	0.40	1.07E−47	5497L	*LOC615876*
SCEc	rs110801791	18	57516245	0.13	− 5.66	0.39	1.36E−43	1252 R	*CTU1*
SCEc	rs135253383	18	57520290	0.13	5.65	0.39	1.66E−43	Within	*CTU1*
SCEc	rs136514789	18	57486184	0.16	4.38	0.36	1.81E−32	1330 L	*LOC540297*
SCEc	rs132734257	18	57511637	0.19	− 4.05	0.34	1.21E−30	5860 R	*CTU1*
SCEc	rs137554975	18	56944300	0.15	− 4.21	0.35	1.42E−30	Within	*MYH14*
SCEc	rs136113894	18	56959011	0.16	− 4.07	0.35	3.22E−29	Within	*KCNC3*
SCEc	rs135508298	18	59602905	0.21	3.90	0.34	7.48E−29	75902 L	*ENSBTAG00000048191*
SCEc	rs134539659	18	59612379	0.21	− 3.90	0.34	7.48E−29		
SCEh	rs109478645	18	57589121	0.12	− 6.13	0.46	4.12E−37	5484 L	*LOC100138951*
SCEh	rs136283363	18	57548213	0.12	6.13	0.46	4.12E−37	5497L	*LOC615876*
SCEh	rs110801791	18	57516245	0.13	− 5.42	0.44	4.98E−32	1252 R	*CTU1*
SCEh	rs135253383	18	57520290	0.13	5.38	0.44	1.17E−31	Within	*CTU1*
SCEh	rs136514789	18	57486184	0.16	4.39	0.40	3.24E−26	1330 L	*LOC540297*
SCEh	rs132734257	18	57511637	0.19	− 4.03	0.39	2.30E−24	5860 R	*CTU1*
SCEh	rs109882115	18	58067310	0.28	− 3.53	0.34	1.33E−23	Within	*ENSBTAG00000039491*
SCEh	rs135508298	18	59602905	0.21	3.90	0.39	5.03E−23	75902 L	*ENSBTAG00000048191*
SCEh	rs134539659	18	59612379	0.21	− 3.90	0.39	5.03E−23		
SCEh	rs43038601	18	57491196	0.22	− 3.59	0.36	4.60E−22	4128 R	*KLK14*
SCSc	rs109478645	18	57589121	0.12	− 5.59	0.82	1.50E−11	5484 L	*LOC100138951*
SCSc	rs136283363	18	57548213	0.12	5.59	0.82	1.50E−11	5497L	*LOC615876*
SCSc	rs136113894	18	56959011	0.16	− 4.41	0.71	9.17E−10	Within	*KCNC3*
SCSc	rs110801791	18	57516245	0.13	− 4.73	0.79	3.63E−09	1252 R	*CTU1*
SCSc	rs135253383	18	57520290	0.13	4.73	0.79	3.63E−09	Within	*CTU1*
SCSc	rs134066287	18	62844651	0.23	3.84	0.65	3.70E−09	Within	*FCAR*
SCSc	rs109882115	18	58067310	0.28	− 3.56	0.60	3.96E−09	Within	*ENSBTAG00000039491*
SCSc	rs109844326	18	63415091	0.16	4.19	0.72	9.77E−09	2950 L	*MBOAT7*
SCSc	rs134353106	18	62839617	0.26	3.47	0.61	2.13E−08	5900 L	*KIR2DL5A*
SCSc	rs133544315	18	60019327	0.21	3.74	0.67	2.82E−08	47852 L	*ZNF836*
SCSh	rs109478645	18	57589121	0.12	− 7.95	0.86	1.86E−19	5484 L	*LOC100138951*
SCSh	rs136283363	18	57548213	0.12	7.95	0.86	1.86E−19	5497L	*LOC615876*
SCSh	rs133544315	18	60019327	0.21	5.91	0.71	1.97E−16	47852 L	*ZNF836*
SCSh	rs110801791	18	57516245	0.13	− 6.94	0.83	2.84E−16	1252 R	*CTU1*
SCSh	rs135253383	18	57520290	0.13	6.86	0.83	5.87E−16	Within	*CTU1*
SCSh	rs109540436	18	59890631	0.20	5.76	0.71	1.05E−15	3982 R	*LOC100300095*
SCSh	rs135033267	18	60043680	0.20	− 5.72	0.71	1.98E−15	25523 R	*ENSBTAG00000011844*
SCSh	rs137436636	18	60006650	0.20	5.72	0.71	1.98E−15	35175 L	*ZNF836*
SCSh	rs136514789	18	57486184	0.16	6.02	0.75	3.07E−15	1330 L	*LOC540297*
SCSh	rs136766893	18	58494682	0.26	− 5.02	0.63	6.02E−15	Within	*BOSTAUV1R416*

^a^Traits = evaluated calving traits included calving ability (CA), daughter calving ability (DCA), maternal calving ease at first calving (heifer; CEh), maternal calf survival at first calving (heifer; CSh), maternal calving ease at later calvings (cow; CEc), maternal calf survival at later calvings (cow; CSc), direct calving ease at first calving (heifer; SCEh), direct calf survival at first calving (heifer; SCSh), direct calving ease at later calvings (cow; SCEc), direct calf survival at later calvings (cow; SCSc) and sire conception rate (SCR). Calving performance traits are expressed as relative breeding values with a population average of 100 and standard deviation of 5

^b^RefSNP (rs) = assigned reference SNP (rs) number for a SNP by the National Center for Biotechnology Information

^c^BTA = *Bos taurus* autosomes

^d^Location = gene location (within *L* left or *R* right) from the SNP of interest

In this study, we confirmed the relationship between the protective functions of the immune system and calving ease and survival by the detection of several genes such as *Fc fragment of IgA receptor* (*FCAR*). *FCAR* includes a significant SNP (rs134066287) which is associated with calving ease and survival at first and later calving (Table 2). *FCAR* is also involved in several immune defense processes such as phagocytosis and *Staphylococcus aureus* infection pathways [68]. The expression profile of fetal membranes, myometrium and cervix tissues showed that inflammatory response was associated with labor [69].

### Genome-wide association for body conformation traits

**Fig. 7:**
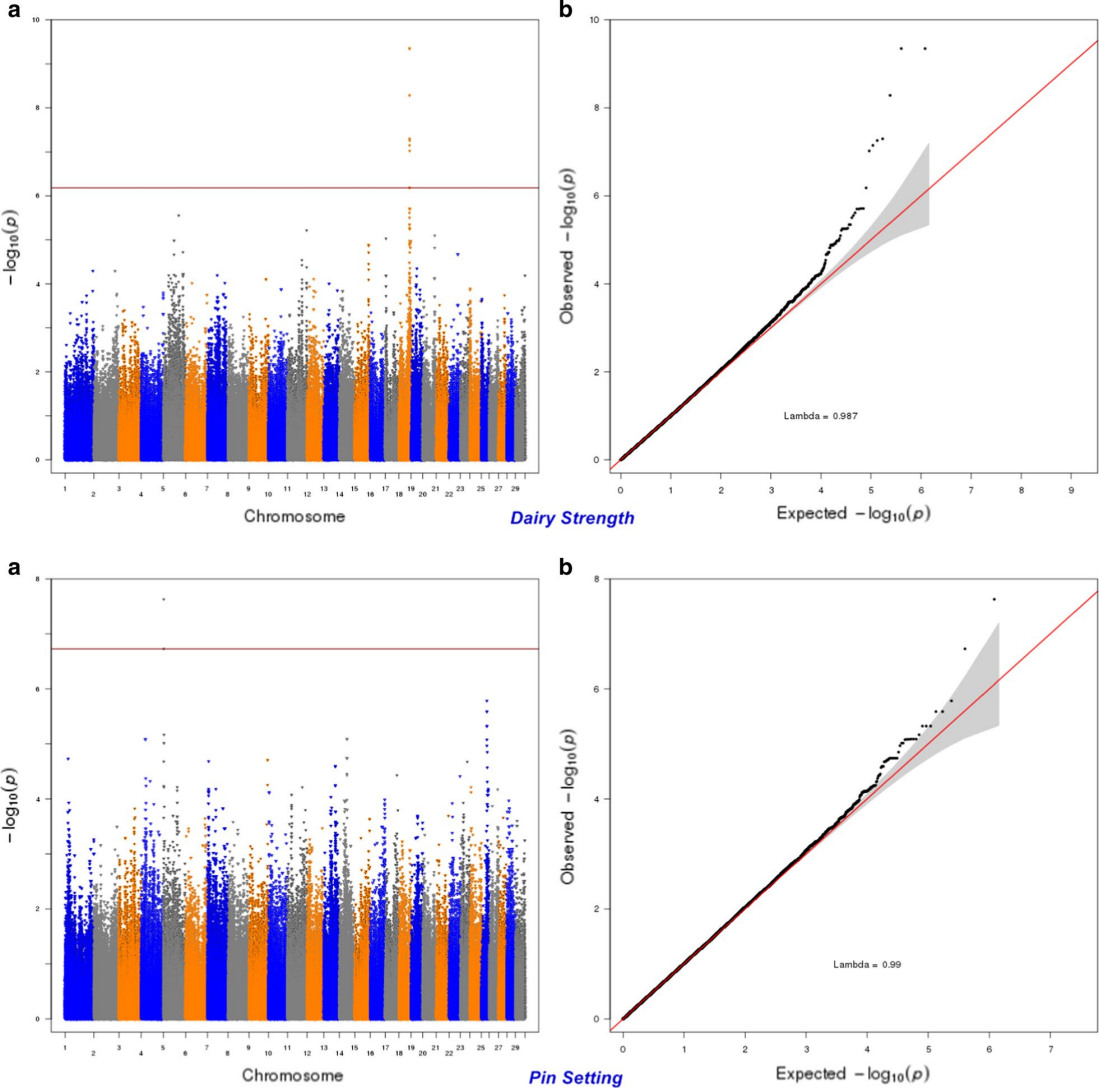
Manhattan (**a**) and Q–Q (**b**) plots from GWAS for dairy strength and pin setting using the Illumina BovineHD BeadChip in Holstein cattle. **a** Manhattan plot in which the genomic coordinates of SNPs are displayed along the horizontal axis, the negative logarithm of the association P-value for each SNP is displayed on the vertical axis, and the dark red line is the significance threshold line at the genome-wise false discovery rate of 5%. **b** Quantile–quantile (QQ) plot showing the late separation between observed and expected values. Genomic inflation factor is around 1 indicating that there is no population stratification

**Fig. 8:**
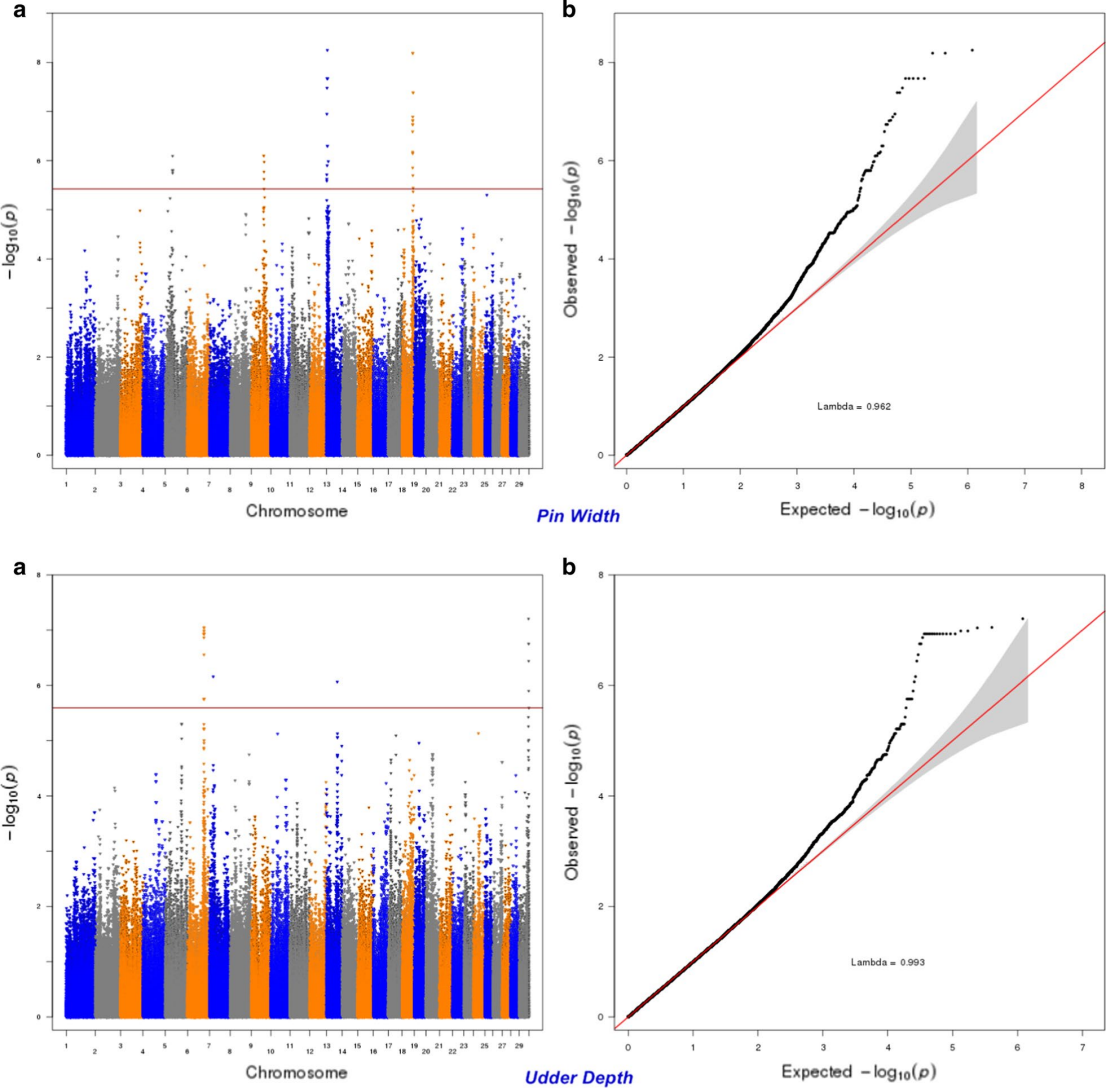
Manhattan (**a**) and Q–Q (**b**) plots from GWAS for pin width and udder depth using the Illumina BovineHD BeadChip in Holstein cattle. **a** Manhattan plot in which the genomic coordinates of SNPs are displayed along the horizontal axis, the negative logarithm of the association P-value for each SNP is displayed on the vertical axis, and the dark red line is the significance threshold line at the genome-wise false discovery rate of 5%. **b** Quantile–quantile (QQ) plot showing the late separation between observed and expected values. Genomic inflation factor is around 1 indicating that there is no population stratification

**Fig. 9:**
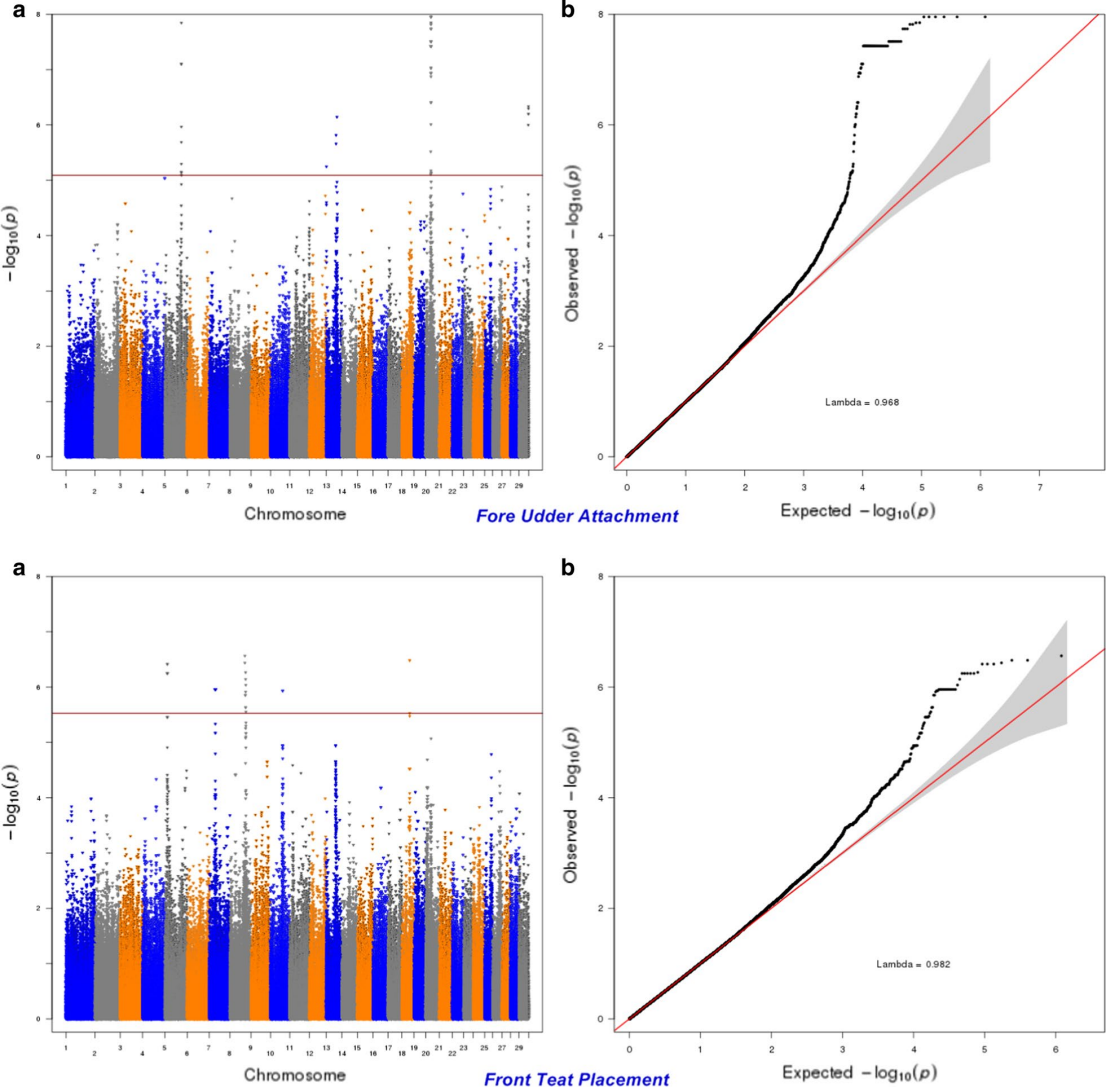
Manhattan (**a**) and Q–Q (**b**) plots from GWAS for fore udder attachment and front teat placement using the Illumina BovineHD BeadChip in Holstein cattle. **a** Manhattan plot in which the genomic coordinates of SNPs are displayed along the horizontal axis, the negative logarithm of the association P-value for each SNP is displayed on the vertical axis, and the dark red line is the significance threshold line at the genome-wise false discovery rate of 5%. **b** Quantile–quantile (QQ) plot showing the late separation between observed and expected values. Genomic inflation factor is around 1 indicating that there is no population stratification

**Fig. 10:**
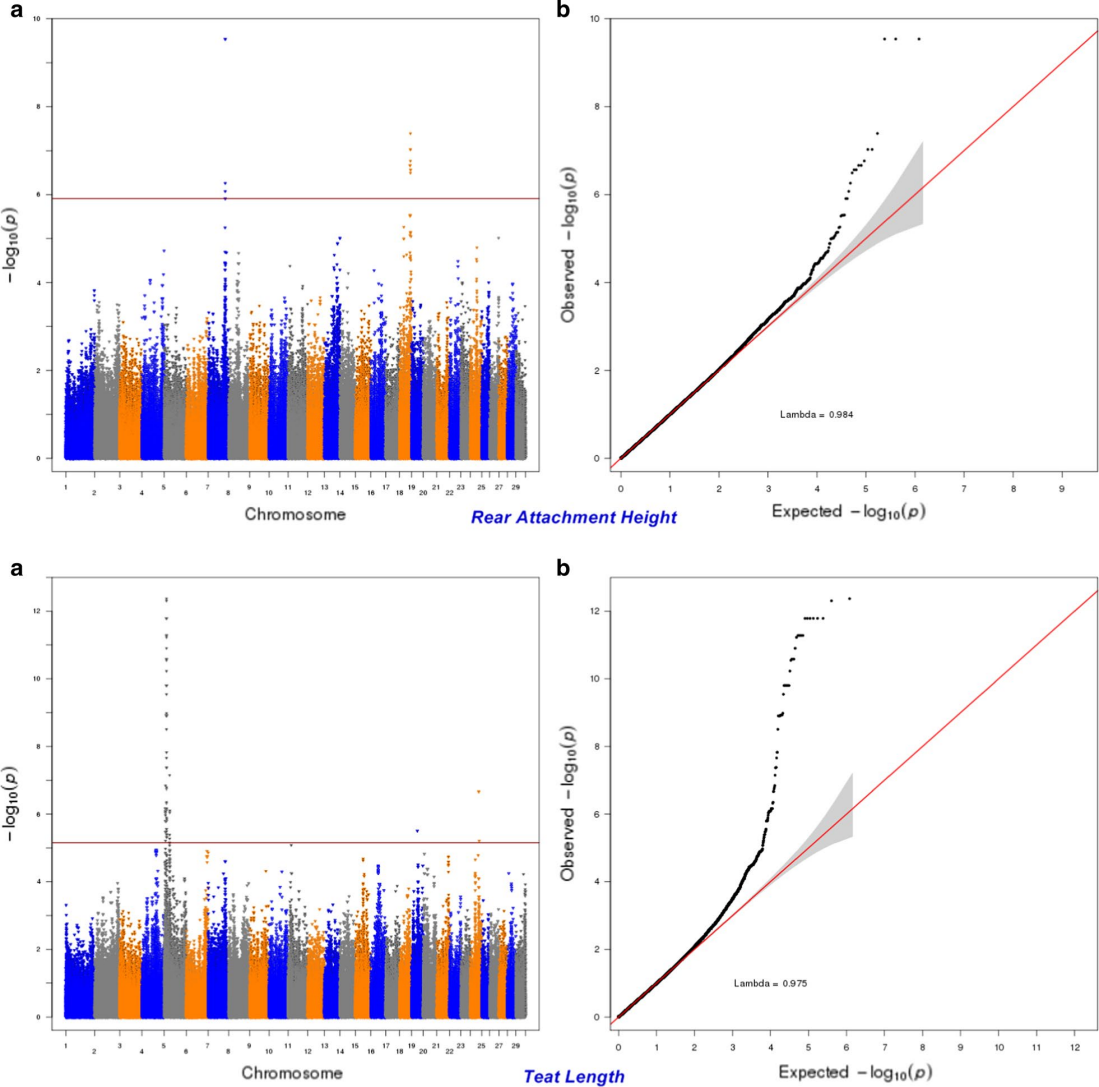
Manhattan (**a**) and Q–Q (**b**) plots from GWAS for rear attachment height and teat length using the Illumina BovineHD BeadChip in Holstein cattle. **a** Manhattan plot in which the genomic coordinates of SNPs are displayed along the horizontal axis, the negative logarithm of the association P-value for each SNP is displayed on the vertical axis, and the dark red line is the significance threshold line at the genome-wise false discovery rate of 5%. **b** Quantile–quantile (QQ) plot showing the late separation between observed and expected values. Genomic inflation factor is around 1 indicating that there is no population stratification

**Fig. 11:**
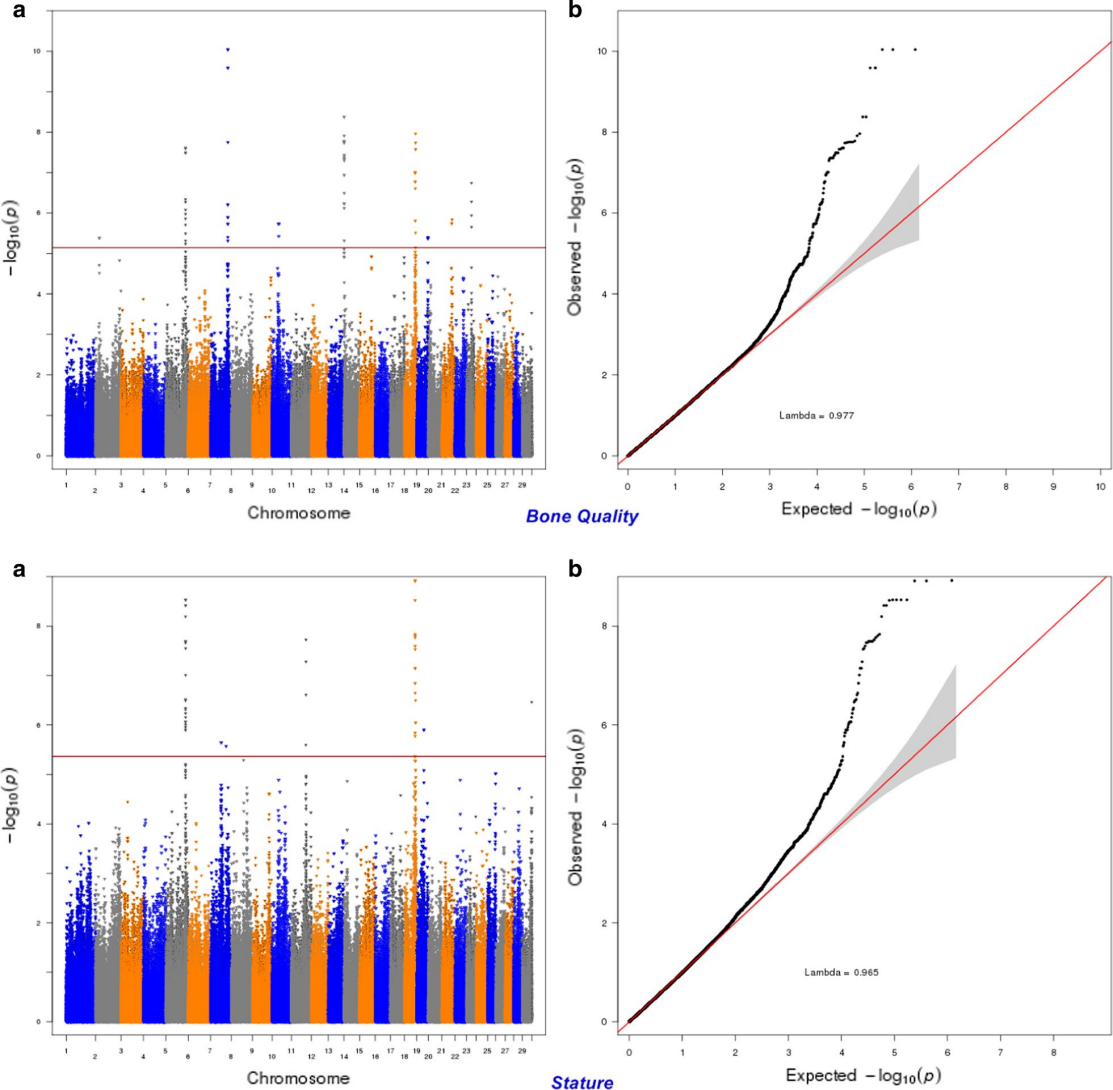
Manhattan (**a**) and Q–Q (**b**) plots from GWAS for bone quality and stature using the Illumina BovineHD BeadChip in Holstein cattle. **a** Manhattan plot in which the genomic coordinates of SNPs are displayed along the horizontal axis, the negative logarithm of the association P-value for each SNP is displayed on the vertical axis, and the dark red line is the significance threshold line at the genome-wise false discovery rate of 5%. **b** Quantile–quantile (QQ) plot showing the late separation between observed and expected values. Genomic inflation factor is around 1 indicating that there is no population stratification

**Fig. 12:**
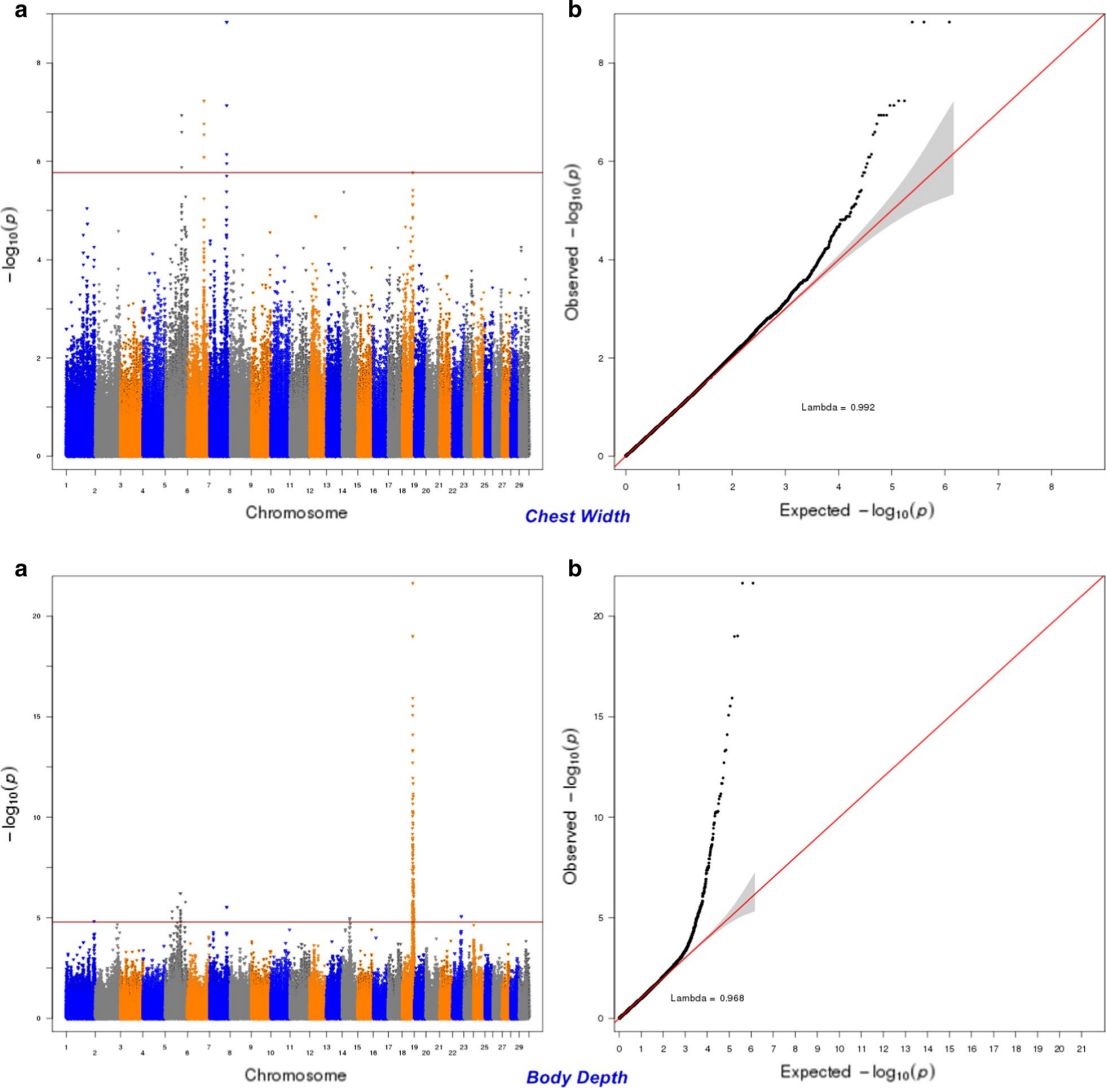
Manhattan (**a**) and Q–Q (**b**) plots from GWAS for chest width and body depth using the Illumina BovineHD BeadChip in Holstein cattle. **a** Manhattan plot in which the genomic coordinates of SNPs are displayed along the horizontal axis, the negative logarithm of the association P-value for each SNP is displayed on the vertical axis, and the dark red line is the significance threshold line at the genome-wise false discovery rate of 5%. **b** Quantile–quantile (QQ) plot showing the late separation between observed and expected values. Genomic inflation factor is around 1 indicating that there is no population stratification

**Fig. 13:**
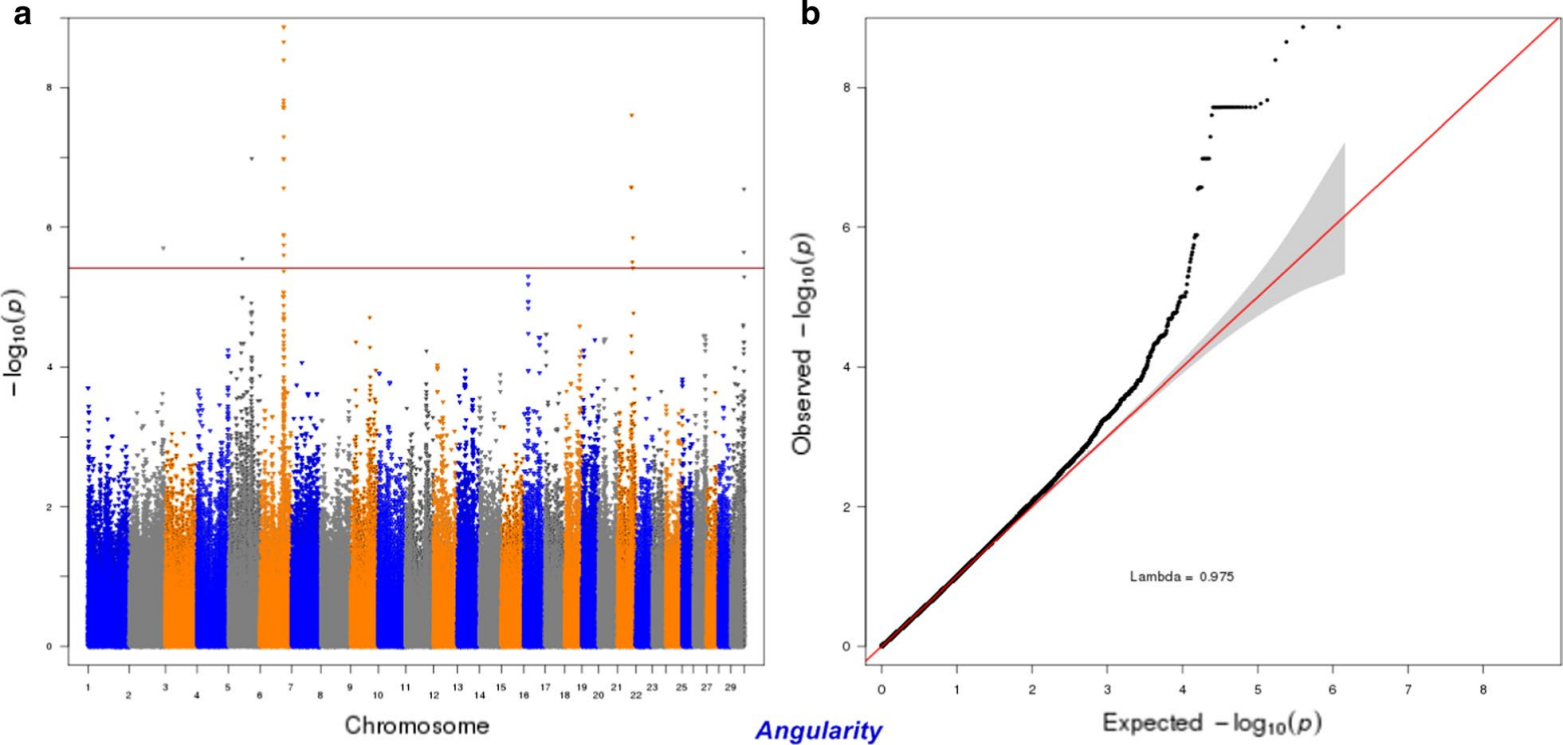
Manhattan (**a**) and Q–Q (**b**) plots from GWAS for angularity using the Illumina BovineHD BeadChip in Holstein cattle. **a** Manhattan plot in which the genomic coordinates of SNPs are displayed along the horizontal axis, the negative logarithm of the association P-value for each SNP is displayed on the vertical axis, and the dark red line is the significance threshold line at the genome-wise false discovery rate of 5%. **b** Quantile–quantile (QQ) plot showing the late separation between observed and expected values. Genomic inflation factor is around 1 indicating that there is no population stratification

**Fig. 14:**
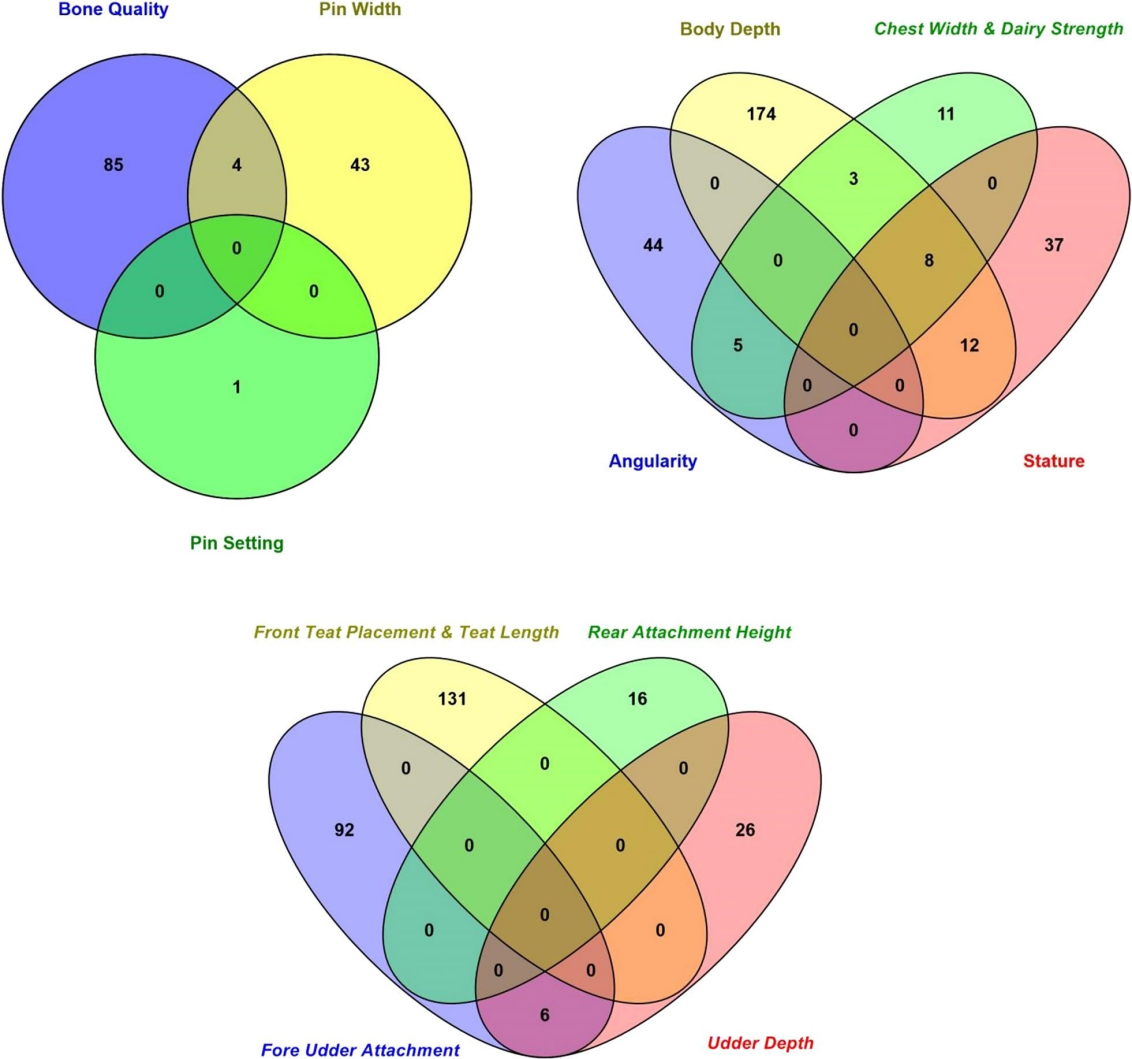
Number of significant (at the genome wise false discovery rate of 5%) SNPs that have a pleiotropic effect on body conformation traits using the Illumina BovineHD BeadChip in Holstein cattle

Six hundred and seven SNPs were statistically significant at a genome-wise FDR of 5% for body conformation traits (Table 3). These significant SNPs were mapped to 553 genes, among which 89 genes included at least one significant SNP and 464 were nearby (100 kbp) one. Peaks indicating association were found on BTA5, 6, 7, 13, 14, 18, and 20 (Figs. 7, 8, 9, 10, 11, 12, 13). Four significant SNPs (FDR of 5%) overlapped between bone quality and pin width (Fig. 14). For example, the *arresting domain*-*containing 3* (*ARRDC3*) gene on BTA7 was associated with body conformation (e.g., bone quality and chest width) and calving performance traits. The *ARRDC3* gene was previously reported as a candidate gene with a pleiotropic effect on birth and weaning weights, direct and maternal calving ease and carcass traits in beef cattle [60]. Furthermore, this study identified other significant polymorphisms for body depth within the *carnitine palmitoyl transferase 1C* (*CPT1C*) gene, which is involved in PPAR signaling, adipocytokine signaling and fatty acid metabolism pathways linked to the animal’s energy balance [70]. Interestingly, we identified a SNP within the *diacylglycerol O*-*acyltransferase 1* (*DGAT1*) gene that was significantly associated with bone quality. Bone quality is an important trait that affects functional longevity of Holstein cows [9]. Kaupe et al. [71] identified a SNP within *DGAT1* that is associated with rump width and strength, which suggests a possible association between *DGAT1* and functional longevity.

**Table 3:** The most highly significant SNPs associated with each body conformation trait at the genome-wise false discovery rate of 5%

Trait^a^	RefSNP (rs)^b^	BTA^c^	Pos. (bp)	MAF	Estimated effect	SE	P-value	Location^d^	Gene name
ANG	rs110434046	6	88919352	0.46829	− 2.46	0.40	1.35E−09		
ANG	rs137712965	6	88922396	0.46829	2.46	0.40	1.35E−09		
ANG	rs136287830	6	88865430	0.46168	− 2.43	0.40	2.21E−09		
ANG	rs108983037	6	88913092	0.46499	2.38	0.40	4.01E−09		
ANG	rs109512265	6	88485244	0.40611	− 2.19	0.38	1.51E−08	Within	*SLC4A4*
BD	rs109478645	18	57589121	0.1195	4.49	0.45	2.29E−22	Within	*ENSBTAG00000037537*
BD	rs136283363	18	57548213	0.1195	− 4.49	0.45	2.29E−22		
BD	rs110801791	18	57516245	0.12539	4.00	0.43	9.73E−20	1252 R	*CTU1*
BD	rs135253383	18	57520290	0.12549	− 3.99	0.43	1.03E−19	Within	*CTU1*
BD	rs132734257	18	57511637	0.19128	3.13	0.37	1.18E−16		
BQ	rs109901274	7	93244933	0.11506	− 2.81	0.43	9.10E−11	Within	*ARRDC3*
BQ	rs109618368	7	93254737	0.11506	2.81	0.43	9.10E−11	1643 L	*ARRDC3*
BQ	rs110680622	7	93287387	0.11506	− 2.81	0.43	9.10E−11		
BQ	rs110066813	7	93251138	0.13045	− 2.52	0.39	2.59E−10	Within	*ARRDC3*
BQ	rs109860522	7	93263102	0.13045	2.52	0.39	2.59E−10		
CW	rs109901274	7	93244933	0.11506	3.10	0.51	1.47E−09	Within	*ARRDC3*
CW	rs109618368	7	93254737	0.11506	− 3.10	0.51	1.47E−09	1643 L	*ARRDC3*
CW	rs110680622	7	93287387	0.11506	3.10	0.51	1.47E−09		
CW	rs110434046	6	88919352	0.46829	2.22	0.41	5.90E−08		
CW	rs137712965	6	88922396	0.46829	− 2.22	0.41	5.90E−08		
DS	rs109478645	18	57589121	0.1195	2.86	0.45	4.48E−10	Within	*ENSBTAG00000037537*
DS	rs136283363	18	57548213	0.1195	− 2.86	0.45	4.48E−10		
DS	rs136514789	18	57486184	0.15844	− 2.29	0.39	5.21E−09	1330 L	*LOC540297*
DS	rs110801791	18	57516245	0.12539	2.39	0.44	5.02E−08	1252 R	*CTU1*
DS	rs134321289	18	58053410	0.31698	− 1.73	0.32	5.49E−08		
FA	rs109517032	20	26955400	0.26761	2.31	0.40	1.11E−08		
FA	rs110521341	20	26957866	0.26761	− 2.31	0.40	1.11E−08		
FA	rs109222733	20	26960799	0.26761	− 2.31	0.40	1.11E−08		
FA	rs109747498	20	26978980	0.26761	− 2.31	0.40	1.11E−08		
FA	rs110486797	20	26990879	0.26761	− 2.31	0.40	1.11E−08		
FTP	rs133797664	8	79527489	0.1322	2.55	0.49	2.73E−07		
FTP	rs136251125	18	42320881	0.29168	1.67	0.32	3.28E−07		
FTP	rs136974352	18	42322043	0.29168	1.67	0.32	3.28E−07		
FTP	rs43571286	8	79497558	0.13468	2.42	0.47	3.66E−07		
FTP	rs137127919	5	12712534	0.06083	2.71	0.53	3.84E−07		
PS	rs42244554	5	1278211	0.3427	2.00	0.36	2.35E−08	694 L	*ZFC3H1*
PW	rs137169221	13	4626355	0.11558	− 2.73	0.46	5.63E−09		
PW	rs109478645	18	57589121	0.1195	2.53	0.43	6.48E−09	Within	*ENSBTAG00000037537*
PW	rs136283363	18	57548213	0.1195	− 2.53	0.43	6.48E−09		
PW	rs42382059	13	4518102	0.1165	− 2.60	0.46	2.12E−08		
PW	rs42382046	13	4524429	0.1165	2.60	0.46	2.12E−08		
RAH	rs109901274	7	93244933	0.11506	− 3.10	0.49	2.88E−10	Within	*ARRDC3*
RAH	rs109618368	7	93254737	0.11506	3.10	0.49	2.88E−10	1643 L	*ARRDC3*
RAH	rs110680622	7	93287387	0.11506	− 3.10	0.49	2.88E−10		
RAH	rs109343241	18	59615343	0.27577	1.93	0.35	4.08E−08		
RAH	rs135508298	18	59602905	0.20564	2.12	0.39	9.37E−08		
ST	rs109882115	18	58067310	0.2799	1.86	0.30	1.19E−09	Within	*ENSBTAG00000039491*
ST	rs109478645	18	57589121	0.1195	2.53	0.41	1.22E−09	Within	*ENSBTAG00000037537*
ST	rs136283363	18	57548213	0.1195	− 2.53	0.41	1.22E−09		
ST	rs133960300	5	106250561	0.14439	− 2.34	0.39	2.94E−09	3346 R	*CCND2*
ST	rs109685956	5	106256616	0.14439	− 2.34	0.39	2.94E−09	Within	*CCND2*
TL	rs110895486	5	12438670	0.21287	3.00	0.41	4.27E−13		
TL	rs109194156	5	12444024	0.21318	2.99	0.41	4.92E−13		
TL	rs110137797	5	12464607	0.21328	2.93	0.41	1.63E−12	Within	*TMTC2*
TL	rs109264225	5	12468154	0.21328	2.93	0.41	1.63E−12	Within	*TMTC2*
TL	rs43435142	5	12468826	0.21328	2.93	0.41	1.63E−12	Within	*TMTC2*
UD	rs42192551	29	49182244	0.46055	− 1.48	0.27	6.23E−08		
UD	rs110432804	6	89036570	0.46932	− 2.00	0.37	8.93E−08		
UD	rs108983037	6	88913092	0.46499	− 2.00	0.37	9.11E−08		
UD	rs110434046	6	88919352	0.46829	2.00	0.37	1.04E−07		
UD	rs137712965	6	88922396	0.46829	− 2.00	0.37	1.04E−07		

Conformation trait EBV are standardized to have a mean value of zero and a standard deviation of 5

^a^Trait = body depth (BD), bone quality (BQ), chest width (CW), pin width (PW), rear attachment height (RAH), stature (ST), angularity (ANG), body depth (BD), dairy strength (DS), fore udder attachment (FA), front teat placement (FTP), pin setting (PS), rear attachment height (RAH), teat length (TL)

^b^RefSNP (rs) = Assigned Reference SNP (rs) number for a SNP by the National Center for Biotechnology Information

^c^BTA = *Bos taurus* autosomes

^d^Location = gene location (within *L* left or *R* right) from the SNP of interest

### Pleiotropic polymorphisms for calving performance and body conformation traits

**Fig. 15:**
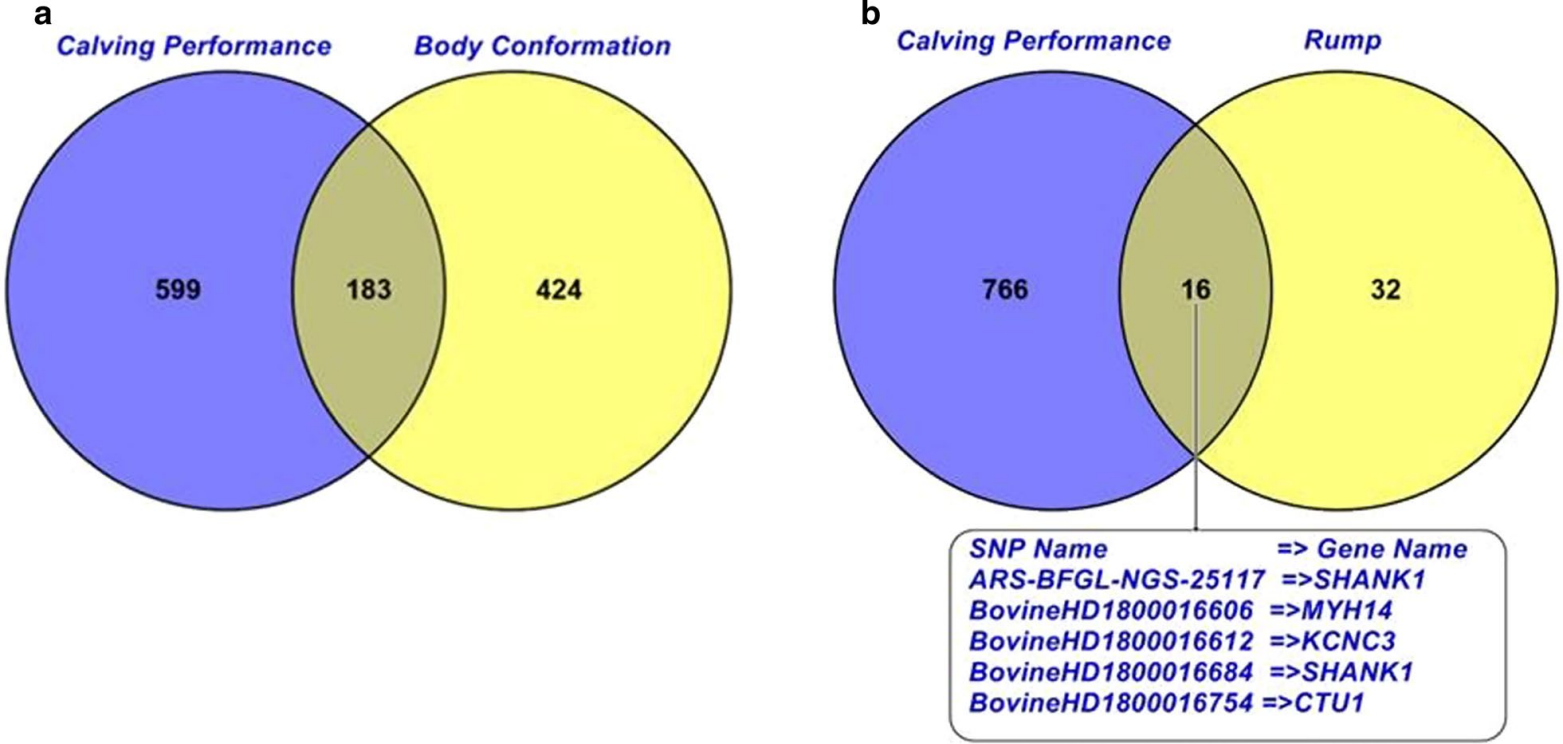
Number of significant (at the genome-wise false discovery rate of 5%) SNPs that have a pleiotropic effect on calving performance and body conformation traits using the Illumina BovineHD BeadChip in Holstein cattle. Rump is the rump traits including pin setting and pin width

One hundred and eighty-three SNPs were associated with calving and conformation traits. Sixteen SNPs on BTA18 in the region between 56.9 and 59.9 Mbp were associated with calving performance and rump traits particularly pin width (Fig. 15). This region harbours the *SH3 and multiple ankyrin repeat domains 1* (*SHANK1*) gene, the *myosin heavy chain 14* (*MYH14*) gene, and the *cytosolic thiouridylase subunit 1* (*CTU1*) gene, which are involved in tight junction, viral myocarditis, regulation of actin cytoskeleton, glutamatergic synapse, and sulfur relay system pathways [72]. The *MYH14* gene is associated with calving and body conformation and with one of the most significant SNPs (rs137554975) identified for eight calving traits. *MYH14* is also involved in the regulation of actin cytoskeleton and tight junction pathways which are critical for myometrial functions during parturition [73]. The pleiotropic associations between calving performance and rump traits support the known genetic correlation between characteristics of the pelvis area (e.g., rump traits) and calving ease and calf survival [8]. A cow with a wide pin, long sloping rump, and slight slope from pin bone to thurl is known to be able to calve easily [8, 74]. An unfavorable genetic association between height of pin bones and slope to the cow’s birth canal was observed, which suggests that higher pin bones may result in a tight birth canal causing calving difficulty [75].

### Functional consequences of significant SNPs and in silico functional analyses

Most of the identified SNPs for calving and/or body conformation traits were intergenic (44.4%), intronic (31%), downstream (11.4%), or upstream (9.8%) of a gene. As shown in Table 4, we identified several SNPs with high functional interest (e.g., non-synonymous coding and splice site intronic variants). For instance, a stop-lost mutation was identified within the *HORMA domain containing 2* (*HORMAD2*) gene and was significantly associated with CA, SCEc and SCEh. *HORMAD2* is involved in the M-phase of the mitotic cell cycle and, in humans, a polymorphism within this gene is known to cause meiotic arrest leading to human azoospermia [76].

**Table 4:** Significant SNPs and their genes associated with calving and conformation traits in Holstein cattle and causing potential functional consequences

SNP accession number	BTA^a^	Position (bp)	Consequence^b^	Amino acid change^c^	Entrez gene name	Type traits	Calving traits
rs134519015	5	58464570	NSC	F/S	*LOC782296*	–	CA
rs110862689	5	105933019	NSC	F/L	*AKAP3*	BQ, ST	CA, SCEc, SCEh
rs109901274	7	93244933	NSC	F/S	*ARRDC3*	BD, BQ, CW, RAH	CA, SCEc, SCEh, SCSh
rs134432442	14	1736599	NSC	N/S	*CPSF1*	BQ	–
rs133271979	14	2019390	NSC	I/M	*GRINA*	BQ	–
rs110073506	18	56740979	NSC	Q/P	*LOC511180*	–	CA, SCEc, SCEh
rs136766893	18	58494682	NSC	L/P	*BOSTAUV1R416*	BD, BQ	CA, SCEc, SCEh, SCSc, SCSh
rs43098627	18	58590005	NSC	Y/S	*BOSTAUV1R420*	–	DCA, SCEc
rs110283226	18	60233963	NSC	I/R	*LOC788599*	BD	CA, DCA, SCEc, SCEh, SCSh
rs110104114	18	60342959	NSC	V/G	*ZNF677*	–	CA, SCEh
rs110302968	18	62832344	NSC	S/R	*KIR2DL5A*	–	CA, SCEc, SCEh, SCSc, SCSh
rs132815594	18	62843699	NSC	E/G	*FCAR*	–	CA, SCEc, SCEh, SCSh
rs41902720	18	63588968	NSC	H/R	*BOSTAUV1R427*	–	CA, SCEc, SCEh
rs41898987	18	65754940	NSC	W/G	*ZNF274*	–	SCEc
rs41892719	18	56183207	SSI	–	*LOC785907*	–	CA, SCEc, SCEh
rs41852917	17	71314935	SL	*/Q	*HORMAD2*	–	CA, SCEc, SCEh

Evaluated traits include calving ability (CA), daughter calving ability (DCA), maternal calving ease (heifer; CEh), maternal calf survival at first calving (heifer; CSh), maternal calving ease at later calvings (cow; CEc), maternal calf survival at later calvings (cow; CSc), direct calving ease at first calving (heifer; SCEh), direct calf survival at first calving (heifer; SCSh), direct calving ease at later calvings (cow; SCEc), direct calf survival at later calvings (cow; SCSc) and sire conception rate (SCR)

^a^BTA = *Bos taurus* autosomes

^b^Consequence = non-synonymous coding (NSC), splice site intronic (SSI), stop lost (SL)

^c^Amino acid change = glutamic acids (E), phenylalanine (F), glycine (G), histidine (H), isoleucine (I), leucine (L), methionine (M), asparagine (N), proline (P), glutamine (Q), arginine (R), serine (S), valine (V), tryptophan (W), tyrosine (Y)

The SNP enrichment analyses provided potential GO terms for calving and conformation traits including biological mechanisms, molecular function and cellular component (see Additional file 6: Table S2). Biological processes including musculoskeletal movement (GO:0050881), meiotic cell cycle (GO:0051321), oocyte maturation (GO:0001556), protein localization to synapse (GO:0035418) and skeletal muscle contraction (GO:0003009) were enriched (FDR of 1%) for calving ease and survival traits. Also, biological processes such as steroid dehydrogenase activity (GO:0016229) and 3-beta-hydroxy-delta5-steroid dehydrogenase activity (GO:0016229) were over-represented (FDR of 1%) for calving ease in heifers. The steroids are very important for the control of the synthesis of oxytocin receptors and can facilitate the initiation of parturition [62].

Thirty-three genes were enriched (P < 0.10) in 13 pathways, in which tight junction, oxytocin signaling and MAPK signaling are associated with calving performance traits (Table 5). The tight junction proteins are highly expressed in the final stages of cervical ripening and dilation in preparation for parturition [76, 77]. Furthermore, mitogen-activated protein kinase (MAPK) signaling pathway was enriched (P = 0.065) based on six genes. MAPK signaling cascades are critical in the regulation of several mechanisms including uterine contractility and myometrial cell proliferation [73]. In agreement with our findings, previous GWAS in Angus and Hereford cattle detected the MAPK signaling pathway as having a pleiotropic effect on birth weight, calving ease (direct and maternal) [60]. In addition, oxytocin signaling pathway which comprises the main drivers of calving [77] was also enriched (P < 0.05) for five genes (see Additional file 7: Figure S5).

**Table 5:** KEGG biological pathways enriched with the identified candidate genes for calving performance and body conformation traits

Pathway name	P-value	Gene name
*Calving performance*		
Tight junction	0.016	*MYH13, MYH14, PPP2R1A, PRKCG, MYH10*
Oxytocin signaling	0.040	*CACNG6, CACNG7, KCNJ14, PPP1R12C, PRKCG*
MAPK signaling	0.065	*CACNG6, FGF21, CACNG7, FGF6, FGF23, PRKCG*
*Body conformation*		
Glutamatergic synapse	0.013	*SHANK1, CACNA1C, PLCB1, SLC17A7, ITPR2*
MAPK signaling	0.017	*FGF21, CACNA1C, FGF23, RRAS, LOC615727, RASGRF2, NTRK2*
Aldosterone synthesis and secretion	0.026	*ATF1, CACNA1C, PLCB1, ITPR2*
Oxytocin signaling	0.035	*OXT, CACNA1C, PLCB1, KCNJ14, ITPR2*
Retrograde endocannabinoid signaling	0.047	*CACNA1C, PLCB1, SLC17A7, ITPR2*
Cholinergic synapse	0.059	*CACNA1C, PLCB1, KCNJ14, ITPR2*
Staphylococcus aureus infection	0.072	*FCAR, C2, CFB*
Dopaminergic synapse	0.077	*CACNA1C, PLCB1, PPP2R1A, ITPR2*
Long-term depression	0.082	*PLCB1, PPP2R1A, ITPR2*
Ribosome	0.090	*RPS11, RPS9, RPL36, RPL13A*
Long-term potentiation	0.096	*CACNA1C, PLCB1, ITPR2*
Renin secretion	0.099	*CACNA1C, PLCB1, ITPR2*

### Validation of genomic predictions

**Fig. 16:**
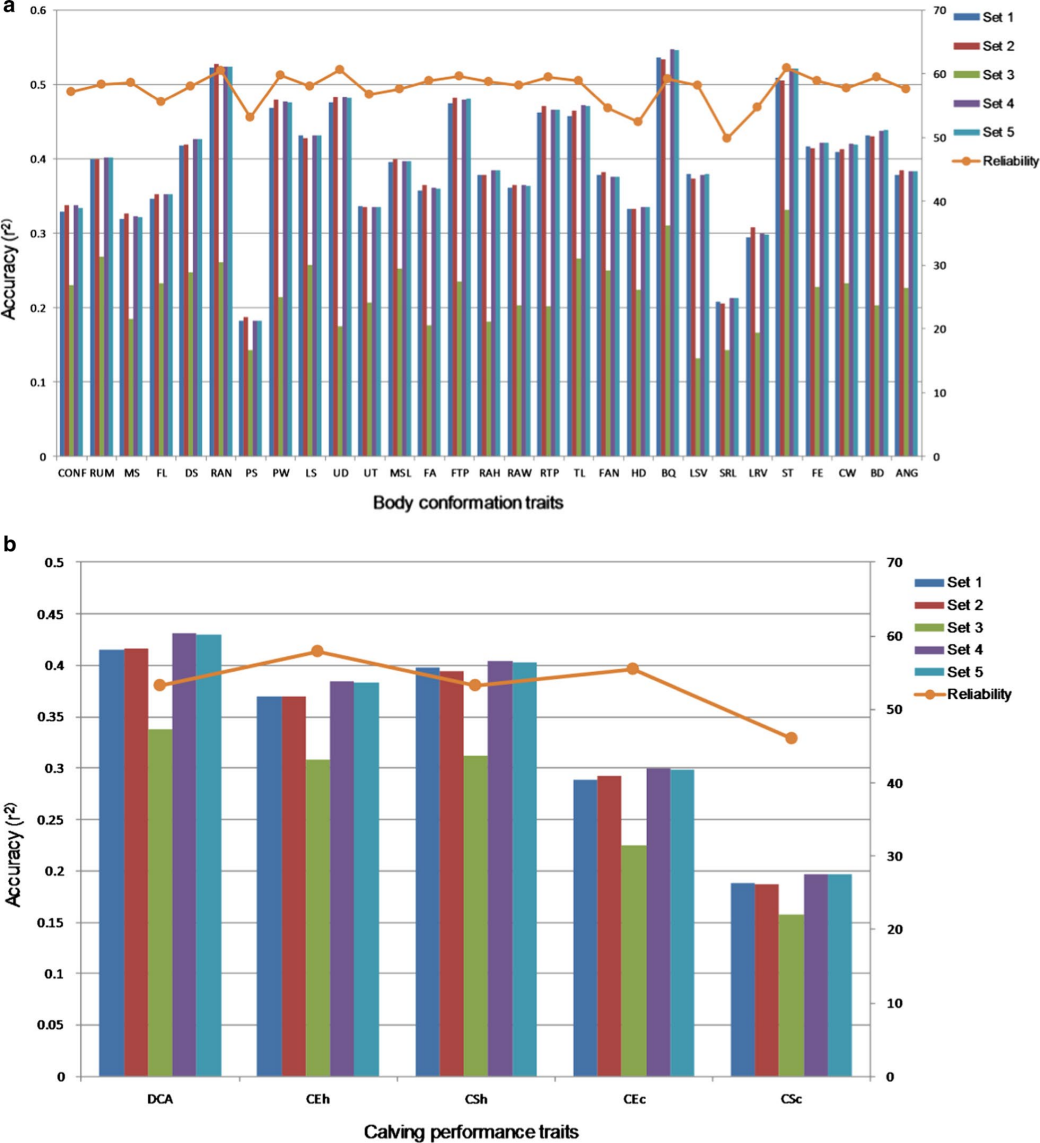
Squared correlation (r^2^) between direct genomic breeding values (DGV) and the corresponding de-regressed EBV for validation bulls using different SNP sets. **a** Calving performance traits are daughter calving ability (DCA); maternal calving ease at first calving (heifer; CEh); maternal calf survival at first calving (heifer; CSh) and maternal calf survival at later calvings (cow; CSc). **b** Body conformation traits are conformation score (CONF); rump (RU); mammary system (MS); feet & legs (FL); dairy strength (DS); rump angle (RAN); pin setting (PS); pin width (PW); loin strength (LS); udder depth (UD); udder texture (UT); median suspensory ligament (MSL); fore udder attachment (FA); front teat placement (FTP); teat length (TL); foot angle (FAN); heel depth (HD); bone quality (BQ); leg side view (LSV); set of rear legs (SRL); leg rear view (LRV); stature (ST); height at front end (FE); chest width (CW); body depth (BD) and angularity (ANG). Set 1 (38,405 SNPs) = set of SNPs from the 50K SNP panel; set 2 (601,717 SNPs) = set of SNPs from the HD SNP panel; set 3 (1206 SNPs) = set of significant (genome-wise false discovery rate of 5%) SNPs for calving and body conformation traits; set 4 (39,564 SNPs) = set of combined SNPs from sets 1 and 3; set 5 (39,408 SNPs) = set of combined SNPs from set 1 and a set of significant SNPs for all traits within or nearby genes (± 100 kbp around the gene); reliability = average reliabilities of the EBV from the December 2014 genetic evaluation for validation population

In this study, there was no substantial difference between prediction accuracies of the DGV from the 50 K or HD panels for young bulls (Fig. 16). This finding is in agreement with the study of Erbe et al. [23], in which the use of 624,213 SNPs provided no extra gain in accuracy than the 50 K panel when using GBLUP in Jersey and Holstein cattle. The reason why the use of both panels yielded similar results may be that the imputed HD panel captures the same loci with a large effect than those captured by the 50 K panel and that other QTL with a small effect captured by the imputed HD panel explain just a small proportion of the genetic variance and, consequently, result in only a small increase in accuracy of prediction of DBV [24]. A slight improvement in the accuracy of DGV when combining the 50 K panel with the significant SNPs or the SNPs located nearby genes compared to using only the SNPs from the 50 K panel may indicate that the HD SNP panel contains SNPs that are potentially linked to causal mutations. However, a decrease of about 6.5% in prediction accuracy on average for calving traits was observed when only significant SNPs (1206) were used compared to the 50 K panel. Nonetheless, the small number of significant SNPs (1206) with a prediction accuracy up to 0.34 supported the informativeness of the candidate genes and the biological mechanisms associated with the traits.

In general, the validation of significantly associated SNPs in a different population is a necessary step before application in breeding programs in order to estimate the accuracy of the DGV properly and to test for potentially spurious associations in the original findings. The calving ability index and direct calving ease and survival traits were not evaluated in the validation analyses because of the limited number of young bulls. The GBLUP method was used because it is the official method for genomic evaluations in Canadian dairy cattle [35], it was recommended by earlier studies [78, 79], and has been shown to be as accurate as other statistical methods [31]. The current study also identified several SNPs with pleiotropic effects, which are associated with known biological pathways involved in calving performance and conformation traits. The correlations between the breeding values of calving performance ranged from 0.11 to 0.82 [80], which suggests that common genes control more than one calving trait, as observed in this study.

## Conclusions

We identified various SNPs that are significantly associated with calving performance on chromosomes 5, 18, and 19 and are located within or nearby genes with potential roles in tight junction, oxytocin signaling, and MAPK signaling. Sixteen SNPs within or nearby the *SHANK1*, *MYH14*, and *CTU1* genes on BTA18 showed a pleiotropic effect on calving performance and body conformation traits. In total, eight, six and four QTL were confirmed for direct calving ease, maternal calving ease, and direct stillbirth, respectively. Combining significant SNPs from the GWAS with SNPs in the 50 K panel for genomic evaluation slightly increased the prediction accuracy of genomic breeding values for calving and body conformation traits. Validation of the SNPs in independent populations, followed by the possible identification of the causal mutations within the validated candidate genes are the logical next research steps.

## Additional files

**Additional file 1: Figure S1.** Distribution of linkage disequilibrium (r^2^) calculated between pairs of SNPs on each chromosome before (A) and after (B) exclusion of the misplaced SNPs. The physical distances between pairs of SNPs are displayed along the horizontal axis, while the r^2^ value for each pair is displayed on the vertical axis. The mean r^2^ over successive intervals of 0.1 Mb is plotted in white

**Additional file 2: Figure S2.** Distribution of 601,717 SNPs in the high-density panel across the bovine genome. Genomic coordinates of SNPs are displayed along the horizontal axis (Mb) and chromosome numbers are displayed on the vertical axis

**Additional file 3: Figure S3.** The first three principal components of genetic co-ancestry based on Illumina BovineHD BeadChip (611,146 SNPs) genotypes for the 4848 bulls in the training population.

**Additional file 4: Figure S4.** Genomic inflation factor (lambda) used for the test statistic (P-value) from the GWAS methods using the generalized linear mixed model accounting for reliability values associated with the de-regressed EBV. The traits are direct calf survival (cow) (SCSc); direct calf survival (heifer) (SCSh); direct calving ease (cow) (SCEc); direct calving ease (heifer) (SCEh); maternal calf survival (cow) (CSc); set of rear legs (SRL): calf survival (heifer) (CSh); daughter calving ability (DCA); heel depth (HD); calving ease (cow) (CEc); pin setting (PS); foot angle (FAN); calving ease (heifer) (CEh); leg rear view (LRV); median suspensory ligament (MSL); udder texture (UT); feet & legs (FL); rear attachment width (RAW); chest width (CW); rump (RU); rear attachment height (RAH); leg side view (LSV); mammary system (MS); loin strength (LS); angularity (ANG); height at front end (FE); conformation (CONF); fore udder attachment (FA); teat length (TL); rear teat placement (RTP); calving ability (CA); bone quality (BQ); front teat placement (FTP); body depth (BD); pin width (PW); dairy strength (DS); rump angle (RAN); udder depth (UD); and stature (ST).

**Additional file 5: Table S1.** List of SNPs associated with calving performance and conformation traits at a FDR of 5% and located within the confidence interval of a previously reported quantitative trait loci (QTL).

**Additional file 6: Table S2.** Gene ontology terms enriched at a FDR of 1% using SNP enrichment analysis for various calving performance and body conformation traits.

**Additional file 7: Figure S5.** The oxytocin signaling pathway enriched for genes (in red boxes) located near the identified significant SNPs for calving performance and body conformation traits.
