# Supplementary material for: Genome-wide association studies and genomic prediction of breeding values for calving performance and body conformation traits in Holstein cattle
Source: Genet Sel Evol. 2017 Nov 7;49:82. doi: 10.1186/s12711-017-0356-8 (PMC6389134; doi:10.1186/s12711-017-0356-8)

**Additional File 1.** **The distribution of linkage disequilibrium (r^2^) calculated between SNP pairs within each chromosome before (A) and after (B) the exclusion of the misplaced SNPs.** The physical distances between SNP pairs are displayed along the horizontal axis, while the r^2^ value for each pair is displayed on the vertical axis. The mean r^2^ over successive intervals of 0.1 Mb is plotted in white.

(A)


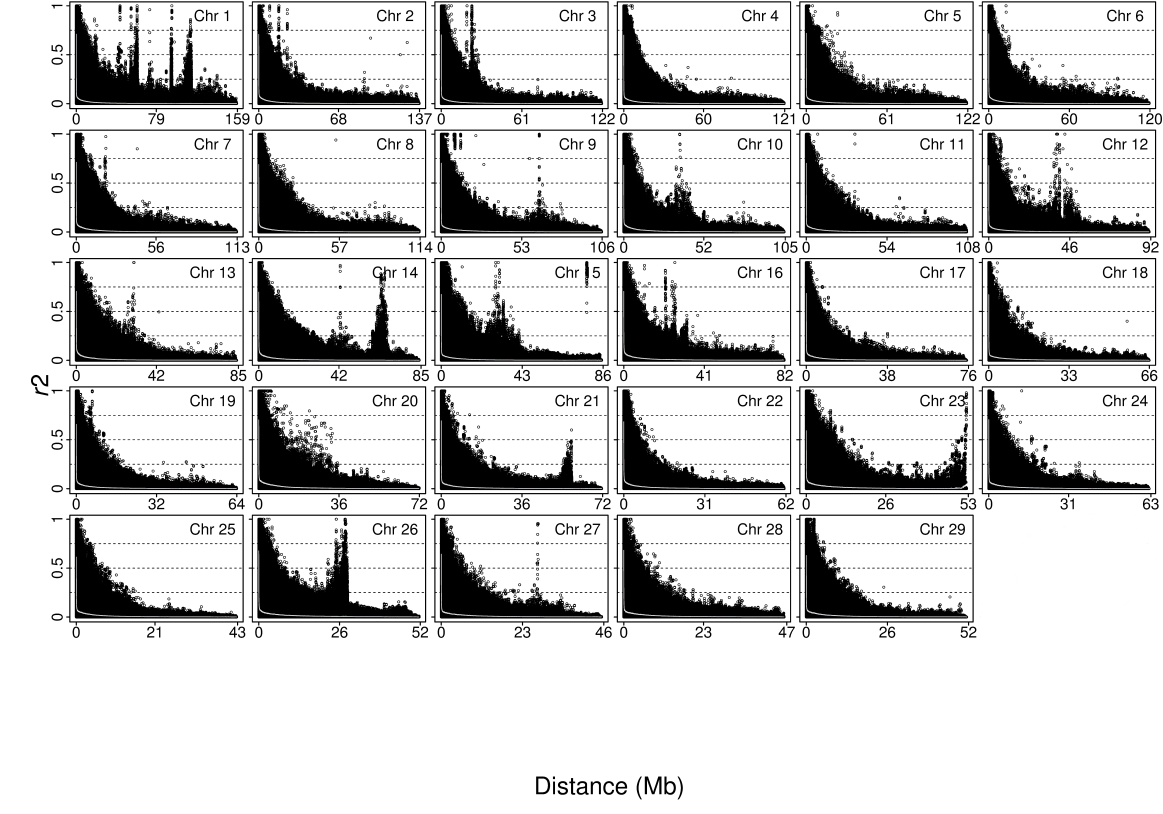


(B)


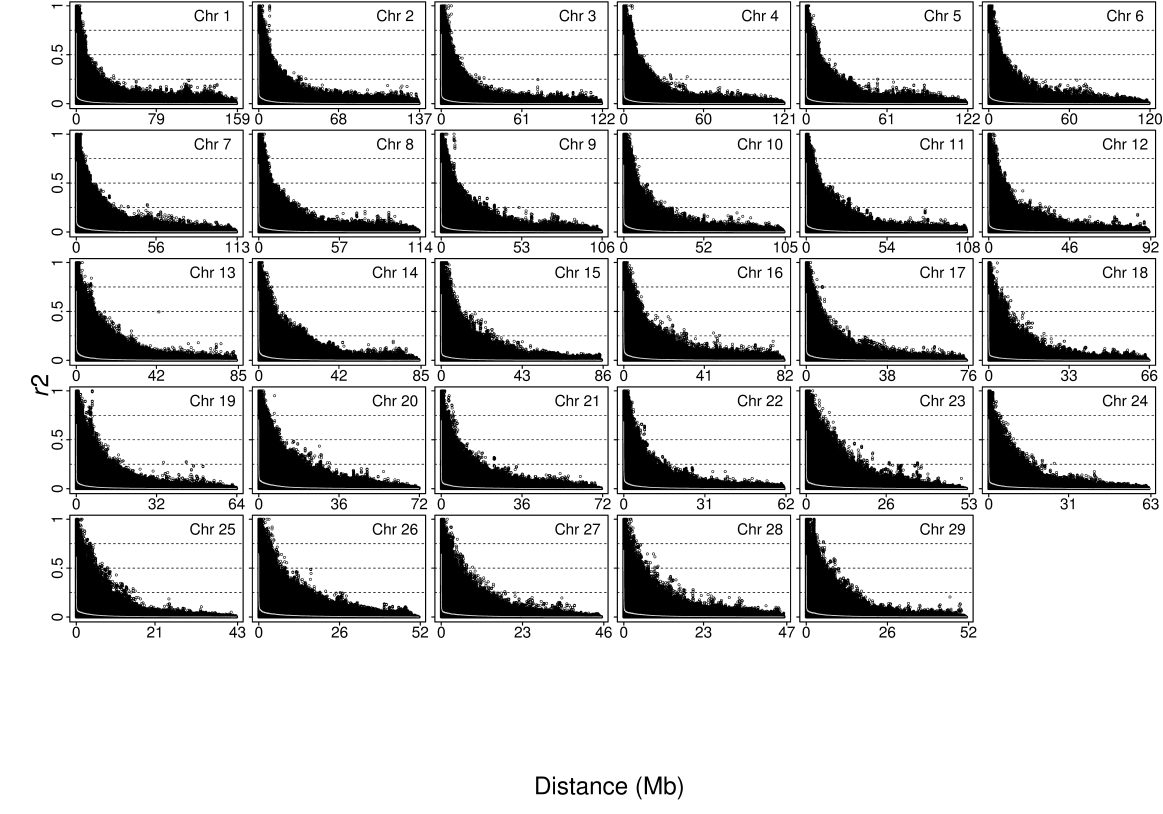

Supplement: Supplementary file 1 — Additional file 1: Figure S1. Distribution of linkage disequilibrium (r2) calculated between pairs of SNPs on each chromosome before (A) and after (B) exclusion of the misplaced SNPs. The physical distances between pairs of SNPs are displayed along the horizontal axis, while the r2 value for each pair is displayed on the vertical axis. The mean r2 over successive intervals of 0.1 Mb is plotted in white [file 12711_2017_356_MOESM1_ESM.docx]
