# Supplementary material for: Genome-wide association studies and genomic prediction of breeding values for calving performance and body conformation traits in Holstein cattle
Source: Genet Sel Evol. 2017 Nov 7;49:82. doi: 10.1186/s12711-017-0356-8 (PMC6389134; doi:10.1186/s12711-017-0356-8)

**Additional file 2.** **The distribution of 601,717 single nucleotide polymorphisms (SNPs) in the high-density panel across the bovine genome.** The genomic coordinates of SNPs are displayed along the horizontal axis (Mb), while the chromosome name is displayed on the vertical axis.


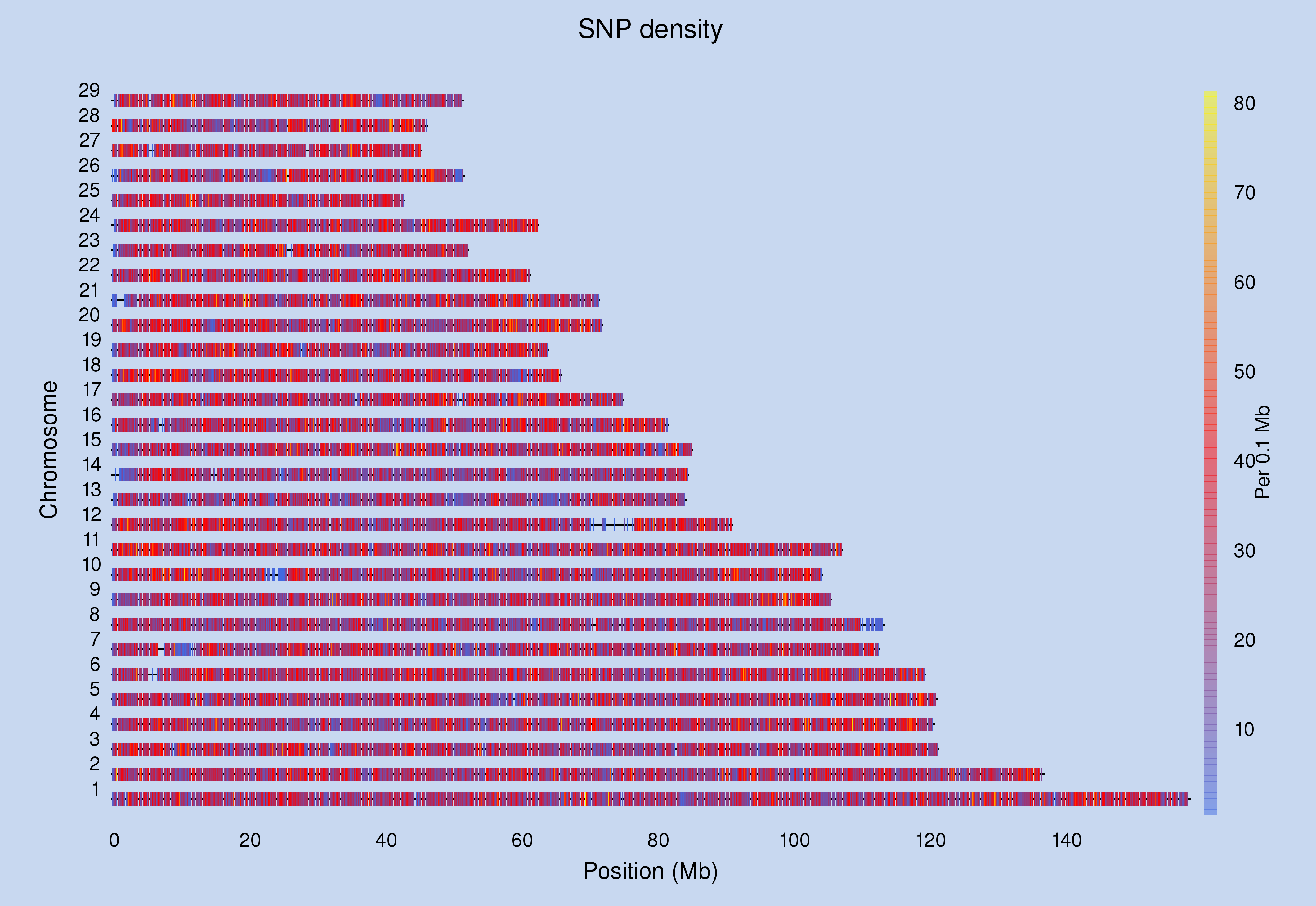

Supplement: Supplementary file 2 — Additional file 2: Figure S2. Distribution of 601,717 SNPs in the high-density panel across the bovine genome. Genomic coordinates of SNPs are displayed along the horizontal axis (Mb) and chromosome numbers are displayed on the vertical axis [file 12711_2017_356_MOESM2_ESM.docx]
