# Supplementary material for: Genome-wide association studies and genomic prediction of breeding values for calving performance and body conformation traits in Holstein cattle
Source: Genet Sel Evol. 2017 Nov 7;49:82. doi: 10.1186/s12711-017-0356-8 (PMC6389134; doi:10.1186/s12711-017-0356-8)

**Additional file 3.** The first three principal components of genetic coancestry based on 611,146 SNPs from Illumina BovineHD BeadChip genotypes on 4,848 bulls in the estimation population.


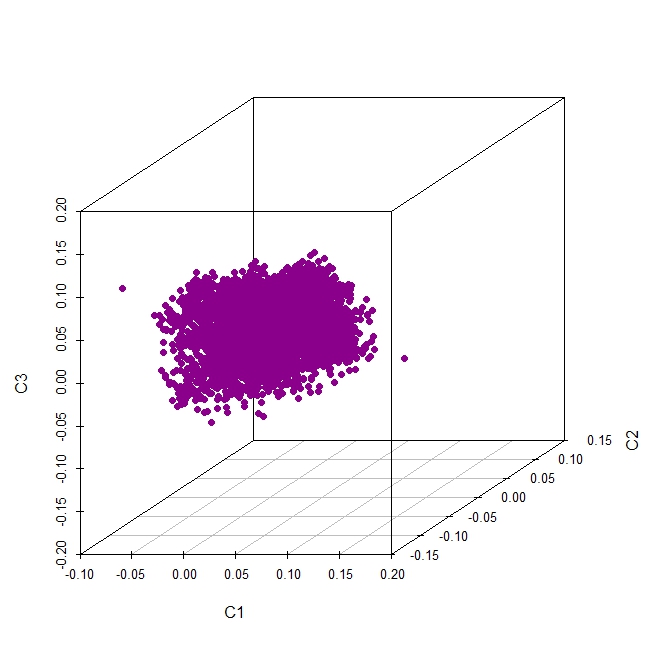

Supplement: Supplementary file 3 — Additional file 3: Figure S3. The first three principal components of genetic co-ancestry based on Illumina BovineHD BeadChip (611,146 SNPs) genotypes for the 4848 bulls in the training population. [file 12711_2017_356_MOESM3_ESM.docx]
