# Supplementary material for: Genome-wide association studies and genomic prediction of breeding values for calving performance and body conformation traits in Holstein cattle
Source: Genet Sel Evol. 2017 Nov 7;49:82. doi: 10.1186/s12711-017-0356-8 (PMC6389134; doi:10.1186/s12711-017-0356-8)

**Additional file 4.** The genomic inflation factor (Lambda) for the test statistic (P-value) from the GWAS methods using generalized linear mixed model accounting for reliability values associated with the de-regressed proofs. The traits are direct calf survival (cow; SCSc); direct calf survival (heifer; SCSh); direct calving ease (cow; SCEc); direct calving ease (heifer; SCEh); maternal calf survival (cow; CSc); set of rear legs (SRL): calf survival (heifer) (CSh); daughter calving ability (DCA); heel depth (HD); calving ease (cow) (CEc); pin setting (PS); foot angle (FAN); calving ease (heifer) (CEh); leg rear view (LRV); median suspensory ligament (MSL); udder texture (UT); feet & legs (FL); rear attachment width (RAW); chest width (CW); rump (RU); rear attachment height (RAH); leg side view (LSV); mammary system (MS); loin strength (LS); angularity (ANG); height at front end (FE); conformation (CONF); fore udder attachment (FA); teat length (TL); rear teat placement (RTP); calving ability (CA); bone quality (BQ); front teat placement (FTP); body depth (BD); pin width (PW); dairy strength (DS); rump angle (RAN); udder depth (UD); and stature (ST).


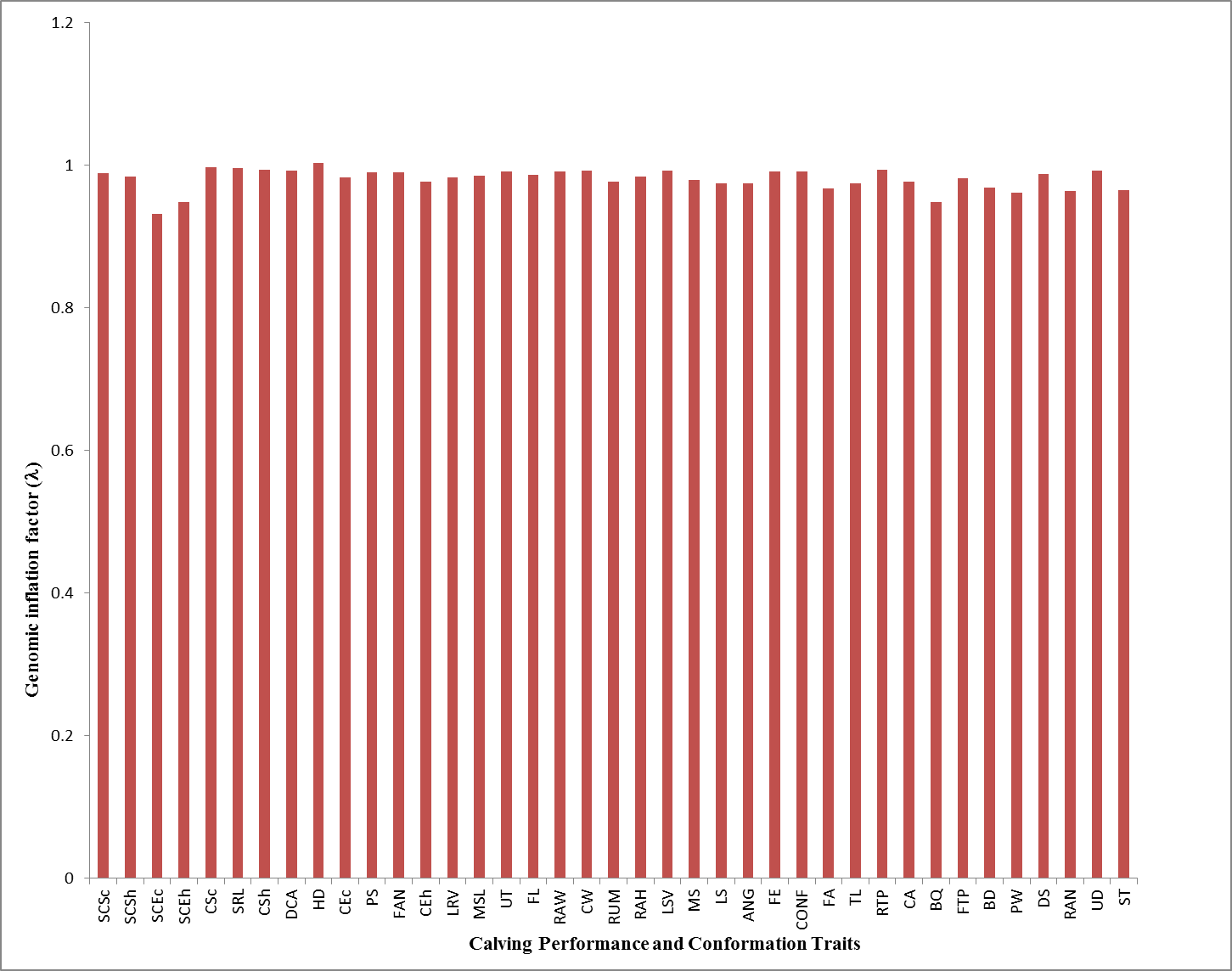

Supplement: Supplementary file 4 — Additional file 4: Figure S4. Genomic inflation factor (lambda) used for the test statistic (P-value) from the GWAS methods using the generalized linear mixed model accounting for reliability values associated with the de-regressed EBV. The traits are direct calf survival (cow) (SCSc); direct calf survival (heifer) (SCSh); direct calving ease (cow) (SCEc); direct calving ease (heifer) (SCEh); maternal calf survival (cow) (CSc); set of rear legs (SRL): calf survival (heifer) (CSh); daughter calving ability (DCA); heel depth (HD); calving ease (cow) (CEc); pin setting (PS); foot angle (FAN); calving ease (heifer) (CEh); leg rear view (LRV); median suspensory ligament (MSL); udder texture (UT); feet & legs (FL); rear attachment width (RAW); chest width (CW); rump (RU); rear attachment height (RAH); leg side view (LSV); mammary system (MS); loin strength (LS); angularity (ANG); height at front end (FE); conformation (CONF); fore udder attachment (FA); teat length (TL); rear teat placement (RTP); calving ability (CA); bone quality (BQ); front teat placement (FTP); body depth (BD); pin width (PW); dairy strength (DS); rump angle (RAN); udder depth (UD); and stature (ST). [file 12711_2017_356_MOESM4_ESM.docx]
