# Supplementary material for: Genome-wide association studies and genomic prediction of breeding values for calving performance and body conformation traits in Holstein cattle
Source: Genet Sel Evol. 2017 Nov 7;49:82. doi: 10.1186/s12711-017-0356-8 (PMC6389134; doi:10.1186/s12711-017-0356-8)

**Additional file 7**. The oxytocin signaling pathway enriched for genes (in red boxes) nearby identified significant SNPs for calving performance and body conformation traits.


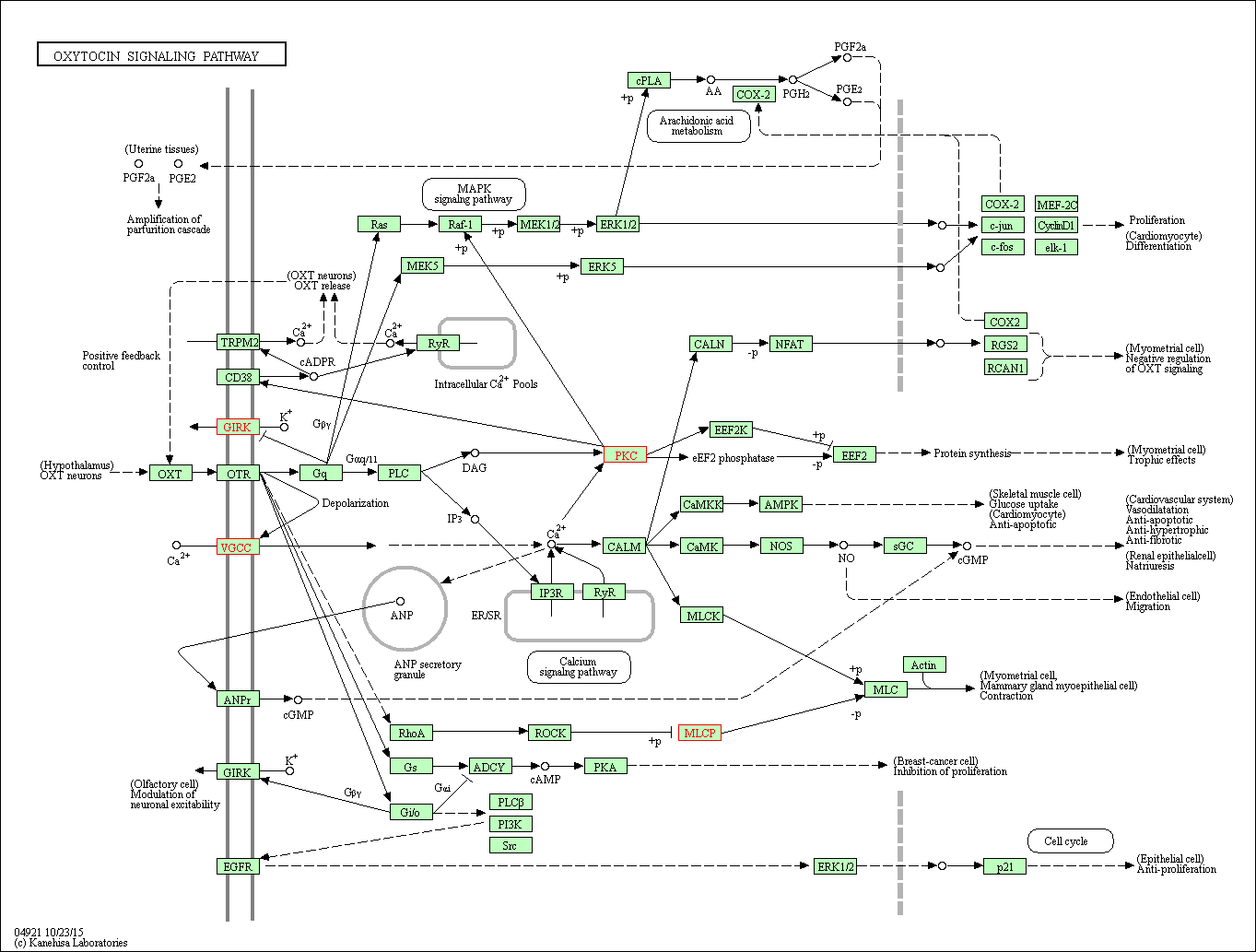

Supplement: Supplementary file 7 — Additional file 7: Figure S5. The oxytocin signaling pathway enriched for genes (in red boxes) located near the identified significant SNPs for calving performance and body conformation traits. [file 12711_2017_356_MOESM7_ESM.docx]
